# Mediating effect of pubertal stages on the family environment and neurodevelopment: An open-data replication and multiverse analysis of an ABCD Study^®^

**DOI:** 10.1016/j.ynirp.2022.100133

**Published:** 2022-09-18

**Authors:** Michael I. Demidenko, Dominic P. Kelly, Felicia A. Hardi, Ka I. Ip, Sujin Lee, Hannah Becker, Sunghyun Hong, Sandra Thijssen, Monica Luciana, Daniel P. Keating

**Affiliations:** a Department of Psychology, University of Michigan, Ann Arbor, MI, USA; b Department of Psychology, Yale University, New Haven, CT, USA; c Behavioural Science Institute, Radboud University, Nijmegen, the Netherlands; d Department of Psychology, University of Minnesota, Minneapolis, MN, USA; e Institute for Social Research, University of Michigan, Ann Arbor, MI, USA

**Keywords:** Pubertal development, Environment, Structural MRI, Resting state MRI, Youth

## Abstract

Increasing evidence demonstrates that environmental factors meaningfully impact the development of the brain (Hyde et al., 2020; McEwen and Akil, 2020). Recent work from the Adolescent Brain Cognitive Development (ABCD) Study^®^ suggests that puberty may indirectly account for some association between the family environment and brain structure and function (Thijssen et al., 2020). However, a limited number of large studies have evaluated what, how, and why environmental factors impact neurodevelopment. When these topics are investigated, there is typically inconsistent operationalization of variables between studies which may be measuring different aspects of the environment and thus different associations in the analytic models. Multiverse analyses (Steegen et al., 2016) are an efficacious technique for investigating the effect of different operationalizations of the same construct on underlying interpretations. While one of the assets of Thijssen et al. (2020) was its large sample from the ABCD data, the authors used an early release that contained 38% of the full ABCD sample. Then, the analyses used several ‘researcher degrees of freedom’ (Gelman and Loken, 2014) to operationalize key independent, mediating and dependent variables, including but not limited to, the use of a latent factor of preadolescents’ environment comprised of different subfactors, such as parental monitoring and child-reported family conflict. While latent factors can improve reliability of constructs, the nuances of each subfactor and measure that comprise the environment may be lost, making the latent factors difficult to interpret in the context of individual differences. This study extends the work of Thijssen et al. (2020) by evaluating the extent to which the analytic choices in their study affected their conclusions. In Aim 1, using the same variables and models, we replicate findings from the original study using the full sample in Release 3.0. Then, in Aim 2, using a multiverse analysis we extend findings by considering nine alternative operationalizations of family environment, three of puberty, and five of brain measures (total of 135 models) to evaluate the impact on conclusions from Aim 1. In these results, 90% of the directions of effects and 60% of the *p*-values (e.g. *p* > .05 and *p* < .05) across effects were comparable between the two studies. However, raters agreed that only 60% of the effects had replicated. Across the multiverse analyses, there was a degree of variability in beta estimates across the environmental variables, and lack of consensus between parent reported and child reported pubertal development for the indirect effects. This study demonstrates the challenge in defining which effects replicate, the nuance across environmental variables in the ABCD data, and the lack of consensus across parent and child reported puberty scales in youth.

## 1. Introduction

Over the last decade, advances in developmental neuroscience have increased our understanding of the effects of environmental experiences on cognitive and emotional development. Empirical evidence from both animal and human literatures (Bick and Nelson, 2016; Farah, 2017; Hackman et al., 2010; Hanson et al., 2013; McEwen and Akil, 2020; Pizzagalli, 2014) demonstrate that developmental changes in the brain reflect responses and adaptations to stressful environmental conditions. These changes have implications for psychopathology (Hyde et al., 2020), health risk behaviors (Duffy et al., 2018) and public policies relating to poverty (Farah, 2018). However, the measurement of environmental stress (e.g., early life adversities or stressful family environments) has contributed to debates on whether broad characterizations of environmental stressors by researchers meaningfully relate to individual differences of brain-behavior associations (Smith and Pollak, 2020). Understanding the convergence between broad and specific characterizations in large datasets, such as the Adolescent Brain Cognitive Development (ABCD) Study^®^, is especially important due to a large number of environmental, demographic and brain measures that are accessible (Barch et al., 2018; Casey et al., 2018; Gonzalez et al., 2021; Zucker et al., 2018). Combining these rich measures with methods that assess reasonable variations, such as multiverse analysis (Steegen et al., 2016), may yield critical insights about the overlap between theoretically valid decisions for future neurodevelopmental research of environmental experiences.

One way to evaluate the associations between theoretically valid decisions of environmental experiences and study conclusions about neurodevelopment is by thoughtfully extending previously published work. Thijssen et al. (2020) provided much needed evidence of the associations between environmental experiences, puberty and neurodevelopment in a large sample (N = 3183) of preadolescents from the ABCD study. The authors grounded their work in a strong theoretical framework of a) how stressful family environments have implications for neurodevelopment, and b) how stressful family environments may increase the pace of pubertal development, which may in turn affect changes in the brain. In this work, the family environment was characterized using a higher-order factor that is composed of subfactors and subscales. Common to most research studies (Gelman and Loken, 2014), the authors had to make important decisions in how they operationalized this variable of the family environment, as well as the parental reported puberty scale and different gray matter, white matter tracts and functional coactivation brain measures. These decisions can be considered as ‘researcher degrees of freedom’ (Simmons et al., 2011). While the decisions in Thijssen et al. (2020) were consistent with the theory that motivated the study, other studies measuring environmental experiences in the ABCD study (as we, Demidenko et al. (2021), and others (Gonzalez et al., 2020; Ip et al., 2022; Petrican et al., 2021; Taylor et al., 2020) have done may reasonably impose different decisions that may contribute to different results and conclusions. Thus, given the rich higher-order model in Thijssen et al. (2020), we first perform a replication of the original study using the full baseline data. Second, we evaluate their core analyses using the multiverse (Steegen et al., 2016) and specification curve analyses (Simonsohn et al., 2020) to investigate how the use of different environmental and puberty variables in the ABCD study data may impact interpretations and conclusions pertaining to the investigated brain-behavior associations. While the replication provides evidence of how effects replicate across research teams and different sets of ABCD data, the specification curve provides a reference for how alternative definitions of key variables that represent stressful experiences in the environment may impact the underlying conclusions.

### 1.1. Measurements of family environmental, puberty and brain

As discussed above and in Smith and Pollak (2020), there are complex and equally plausible ways to define a stressful environment. This is especially true given the ecological context in which development occurs (Bronfenbrenner and Morris, 2007; Oshri et al., 2020) and how environmental stressors are linked to the developing brain (Hyde et al., 2020). Research studies may use broad constructs or individual measures to numerically represent environmental experiences. While individual measures may strongly reflect single latent factors, understanding the associations of individual measures in a more nuanced way may be valuable to the meaningful interpretation and comparison of findings.

One way to evaluate environmental experiences is to study the quality of a child’s family environment and its potential, associated stressors. Within the ABCD study design, Thijssen et al. (2020) define the family environment as a construct encapsulating interactions between family members, socioeconomic status (SES) and psychopathology in the home. It is based on the evolutionary theory of psychosocial acceleration (Belsky et al., 1991), whereby children adapt their development based on their environment, such as caregiving, availability of resources and interpersonal relationships. The latent measure of the family environment used in Thijssen et al. (2020) consisted of child-report of parental conflict, monitoring and acceptance, parent-report of conflict and psychopathology variables, and several demographic questionnaires from the ABCD study. This conceptualization of stressful experiences in the environment, which combines a variety of factors into a single measure of family environment, is comparable to the cumulative risk approach (Evans et al., 2013; Smith and Pollak, 2020).

Given that children often encounter a constellation of risk factors and that the cumulative effect of multiple risks is greater than any singular risk combined (Evans et al., 2013; Gach et al., 2018), examining the aggregated impact of multiple risk factors has been a prominent way to study the impact of stressful family environments. Contemporary frameworks have also examined the impact of unique environmental stressors as distinct types of adverse events using a dimensional approach (McLaughlin et al., 2014, 2017, 2021). It is unclear, however, whether different operational approaches (e.g., lumping or splitting) when measuring the family environment may be associated with divergent outcomes (Smith and Pollak, 2020) or lose information about individuals and critical caregiving factors (Pollak and Smith, 2021).

The quality of parenting and resources within an environment are reported as important contributors to socioemotional development, but their associations with neurodevelopment remain unclear. Experiences such as parental separation (Corrás et al., 2017), parent-child and family conflicts (Repetti et al., 2002), or growing up in impoverished environments (Conger et al., 2002; McLoyd, 1998) have been reported to increase risks for child behavioral problems (Flouri et al., 2014) and poor functioning in academic settings (Hair et al., 2015). It is proposed that such adversities may accelerate maturation of subcortical structures that are implicated in emotion regulation (Callaghan and Tottenham, 2016), such as the amygdala (Whittle et al., 2014) and anterior cingulate cortex (ACC; Thijssen et al., 2020; Zuo et al., 2019), in addition to their corresponding functional coactivations (Gee et al., 2013; Park et al., 2018; Thijssen et al., 2017). However, there is nuanced variation among the associations between the family environment and neural structures and function. Many studies, for instance, report that brain volume and surface areas are smaller in children of low SES (Farah, 2017) or those who were exposed to childhood trauma (De Bellis et al., 1999), but larger in other cases (Tooley et al., 2021). This poses the question of how broad (e.g., latent factors) and individual measures (e.g., parental conflicts or income) relate to neurodevelopment. In fact, work by Rakesh et al. (2021a,b) using the ABCD study data reported that different constructs of stressful experiences (i.e., neighborhood disadvantage, parental education and high household income-to-needs ratios) were uniquely related to specific functional networks.

The subtleties of the family environment may be especially important as they relate to the effects of environmental adversities and pubertal onset. For example, adverse family environments have been reported to cause earlier pubertal onset (Belsky, 2019; Ellis and Garber, 2000; Kim and Smith, 1998; Moffitt et al., 1992); however, there are also reports that the effects of early stress on puberty may depend on specific experiences (Colich et al., 2020) and child sensitivity to environmental contexts (Ellis et al., 2011). These inconsistencies may in part be attributed to challenges in measuring pubertal development. For instance, parents and children may have differing accounts of child pubertal development. This lack of concordance may be more pronounced at younger ages, including critical prepubertal stages (Clawson et al., 2020; Kapetanovic and Boson, 2020; Yi-Frazier et al., 2016). For boys, physical changes may be less readily apparent to parents (Dorn et al., 2003). These factors suggest that how pubertal development is measured may introduce bias that could produce differing conclusions about the relations between puberty and environmental adversities.

To date, investigations using the ABCD study data have drawn upon different measures of the family environment and puberty when studying neurodevelopment. For instance, studies focused on neurodevelopment have used *latent factors* of the family environment (Thijssen et al., 2020, 2022), bio-psycho-social ecologies (Gonzalez et al., 2020), neighborhood and family income/stress (Demidenko et al., 2021; Ip et al., 2022; Sripada et al., 2021; Taylor et al., 2020) and material deprivation/threat/social support (DeJoseph et al., 2022; Petrican et al., 2021), or *individual scales* of family-to-needs ratios (Gonzalez et al., 2020; Rakesh et al., 2021), poverty levels (Ellwood-Lowe et al., 2021), parental education (Rakesh et al., 2021a,b), area deprivation indices (Rakesh et al., 2021; Rakesh et al., 2021) and parental acceptance (Rakesh et al., 2021). As for measures of pubertal development (see reviews regarding measures and correspondence of pubertal scales: Cheng et al. (2021) and Herting et al. (2021)), published works using ABCD data have used parent-reported pubertal development (Demidenko et al., 2021; McNeilly et al., 2021; Thijssen et al., 2020, 2022) or youth/parent reported averages of pubertal development (Petrican et al., 2021). Given variations in the analytic choices regarding measures of the family environment and puberty, the overall goal of the current analysis is to use a multiverse approach to understand the nuanced associations among stressful family environmental experiences, puberty and neurodevelopment when using broad measures (i.e., latent factors) that are comprised of multiple scales as well as specific measures (i.e., individual scales) that are based on individual scales.

### 1.2. Evaluating robustness using multiverse analyses

Multiverse analyses (Steegen et al., 2016) have emerged in psychology in response to the replication crisis in the field (Loken and Gelman, 2017; Open Science Collaboration, 2015). In its simplest form, multiverse analyses capture all possible results of analyses stemming from reasonable variations of data preparation and variable selections, such as decisions by researchers to use a median split or latent factor to operationalize a variable, as in the variety of measures of environmental adversities. These decisions may at times be considered as ambiguous and thus categorized as a researcher’s degrees of freedom in the analytic process (Simmons et al., 2011). For instance, decisions may comprise broad or specific operationalizations of constructs intended to capture different forms of environmental experiences (Smith and Pollak, 2020). As the observed data for individuals across different variables varies and statistical models leverage this variability to make statistical inferences, the multiverse allows the comparison of “*many worlds*” of data (Steegen et al., 2016, p. 703) and draws inferences from the *many statistical results*.

The multiverse technique can be used to explore and aggregate how robust an effect is across different measures and model permutations. One approach used to aggregate the results from the multiverse analysis is to use a specification curve (Simonsohn et al., 2020). The specification curve analysis runs all specified model permutations, reporting the range of effects for each model in one panel and the associated variables included in the model for the respective effect in a second panel. This technique provides a visual representation of the variation of positive, negative and null effects and their significance across the range of variables that may have *reasonably* been specified in a model.

Multiverse analyses have been used in behavioral and neurodevelopmental work. For instance, Orben et al. (2019) evaluated the association between social media use and life satisfaction, reporting inconsistencies that were dependent on how sex was modeled and which analytical method used. Bloom et al. (2021) used a multiverse analysis to evaluate the robustness of age-related changes in functional activation and brain connectivity in their longitudinal cohort of 4–22 year-olds. Across their model permutations, they reported age-related associations in functional activation in the amygdala were relatively robust to model permutations but findings for amygdala-medial prefrontal cortex connectivity were inconsistent across model permutations. Finally, Rijnhart et al. (2021) used a multiverse analysis on a mediation model to examine indirect, direct and total effects of models evaluating whether fat mass mediated the association between weight change and bone mineral density. Across their models, they observed effects that were consistent and in agreement with existing work, which suggested that association between weight change and bone mineral density was robust overall, across alternative analytic decisions in their sample. The literature demonstrates the feasibility of multiverse analyses to provide nuanced information regarding how key variables and statistical decisions relate to the consistency of evidence for prevailing hypotheses.

### 1.3. Current study

The current study consists of two aims which attempt to replicate (Aim 1) and extend (Aim 2) the findings from primary analyses in Thijssen et al. (2020) that used the Release 1.1 data from the ABCD study. The focus in this study is on associations between the (a) environmental variables (i.e., parent and youth reported family conflict scale, youth reported parental monitoring and parental acceptance, and parent reported income/education) and (b) structural (T1; Bilateral Amygdala Subcortical, Bilateral Anterior Cingulate Cortex (ACC) Cortical Thickness, Bilateral ACC Cortical Area) and resting state coactivations (e.g., bilateral Cingulo-Opercular Network and Amygdala functional connectivity), and how (c) reported puberty mediates these associations. Given that the focus here is on the primary analysis in Thijssen et al. (2020) and the limited variability on the pubertal scale in baseline release of the ABCD study 9–10 years old sample, we do not evaluate the stratified differences of sex.

In Aim 1, we conduct a *hybrid-replication* of the primary mediation models from Thijssen et al. (2020; which used the partial release, 37%, of the baseline ABCD data) in the full release of the baseline data. Given the definition of reproducibility and replicability in the literature (Artner et al., 2021), we refer to this as hybrid replication because the baseline ABCD data used here comprises part of the original dataset and 63% new data. Based on replication studies (Open Science Collaboration, 2015), we evaluate whether the results replicate by considering three metrics: 1) consistency in direction and significance of the indirect and direct effects in these analyses and the original published work, 2) evaluate whether the estimates from this original study overlapped with the 95% confidence interval for the replication study and 3) a subjective rating of reproducibility of effects by randomly selecting a subset of coauthors to assess whether estimates did or did not replicate.

In Aim 2, we extend Aim 1 findings by using a multiverse analysis that varies along the independent variable (the overarching family environment factor) and the mediator (parent reported, youth reported and youth/parent reported average puberty). Within the constraints of the ABCD study design, we consider the theoretically plausible sub-factors and individual scales that may be used in future research to evaluate the family environment, as well as the parent and youth reported variable of pubertal development. We report the results of the multiverse analysis of the mediation model using specification curves (Rijnhart et al., 2021; Simonsohn et al., 2020) for the direct, indirect and total effects.

Some reasonably assert that, like the definitions in an analysis pipeline, defining the multiverse is often arbitrary (Del Giacco et al., 2021). Hence, we use alternative derivations of the variables that are modeled in the Family Environment factor in Thijssen et al. (2020), such as sub-factors (i.e., Parent, Child and Demographic factors) and individual scales (e.g., Parental monitoring and Parental Income/Education). These derivations have been and may be used in future ABCD studies using theoretical frameworks to obtain numerical representations of stressful experiences in the environment, and to identify differences between the use of parent and youth reported pubertal development. These results will have important implications for replication and variability of effects using different measures.

## 2. Methods

### 2.1. Participants

The ABCD Consortium study includes longitudinal data that are released based on a predefined schedule (https://abcdstudy.org/scientists/data-sharing/). The ABCD sample is composed of 11,878 9- and 10-year-old preadolescents enrolled across 21 ABCD research sites (Garavan et al., 2018; Volkow et al., 2018). The analyses in Thijssen et al. (2020) utilized Data Release 1.1 of the ABCD Study, which represented approximately 37% of those preadolescents. For this data replication and extension study, data are drawn from the Data Release 3.0. Consistent with Thijssen et al. (2020), several exclusion criteria are applied. Participants were excluded if the structural gray matter data was moderately/severely impacted by (1) motion, (2) intensity inhomogeneity, (3) white matter underestimation, (4) pial overestimation, or if the resting state fMRI (5) average framewise displacement value was greater than 0.55mm and (6) a fieldmap was not collected within two scans. Then, also consistent with the original work, participants were excluded if (1) the guardian completing the survey at the visit was not a biological parent (i.e., biological mother or father), (2) participant is a twin/triplet and (3) if siblings were enrolled from a family, one sibling was randomly excluded. This scheme resulted in a sample of 6658 participants of which N = 2482 (37%) were represented in the first partial release. The distinction between the first and subsequent releases is based on the August 30, 2017, data cut-off provided by the ABCD Data Analytics and Resource Center. Code used in these analyses is publicly available (Demidenko, 2022).

### 2.2. Measures

Complete details of the measures used are available in the Supplemental Materials and in Thijssen et al. (2020). Abbreviated information is presented below. For more details about the variables and code covered in this section, please refer to the associated files with this OSF preregistration on GitHub (Demidenko, 2022).

### 2.3. MRI

Consistent with the original paper, we used the tabulated summary statistics of MRI data provided by the ABCD consortium’s data analytic core through their neuroimaging processing algorithm and subject-level analysis plans noted in the Hagler et al. (2019). Specifically, we focus on the Bilateral Amygdala Subcortical Volume, Bilateral ACC Cortical Thickness, Bilateral ACC Cortical Area and resting state coactivations of bilateral Cingulo-Opercular Network and Amygdala functional connectivity. Bilateral values, such as the Bilateral Amygdala Volume, reflect the mean of the left and right structural estimates. The ACC Fractional Anisotropy was excluded in the present analyses, as there were reported preprocessing differences for diffusion weighted data following release 1.1. Part of the results in Thijssen et al. (2020) were later updated due to corrections that were announced by the ABCD consortium regarding the preprocessing of rsfMRI data (Thijssen et al., 2021).

Data were acquired from T1-weighted anatomical scans and resting-state fMRI (see Casey et al. (2018) for more details). Prior to scanning, participants were invited to experience mock scanners to familiarize themselves with MRI procedures. Head motion was monitored while participants were in the MRI scanners and corrected for as part of the analyses. MRI preprocessing and analyses information, which were conducted by the ABCD consortium’s data analytic core is in part summarized in the original publication (Thijssen et al., 2020, 2021) and provided in Hagler et al. (2019).

### 2.4. Environmental measures

#### 2.4.1. Child items/factor

An abbreviated measure of maternal acceptance, the *Child Report of Parent Behavior Inventory* (5-item CRPBI; Schaefer, 1965), is used. Items are averaged such that *higher scores indicate higher perceptions of parental acceptance* (i.e., higher scores are interpreted as a more *positive* family environment). The youth reported *Family Environment Scale* (9-item FES-Y; Moos and Moos, 1976) is a youth self-report measure that assesses family social environment as perceived by the family member. Items are averaged and *reverse* coded such that *higher scores indicate lower perceptions of family conflict* (i.e., higher scores interpreted as a more *positive* family environment). The *Parental Monitoring Survey* (5-item PMON; Chilcoat and Anthony, 1996) is a youth self-report measure that assesses parental monitoring/supervision. Items are averaged such *that higher scores indicate higher parental monitoring* (i.e., higher scores interpreted as a more *positive* family environment).

For the current study, the Child Factor is composed of the individual items of the CRPBI, FES-Y and PMON scales. To account for the reliability of each scale, a confirmatory factor analysis was submitted to *lavaan::cfa()* (Rosseel et al., 2021) in R version 4.0.3 (R Core Team, 2020). Each measure’s items load onto their respective scales (i.e., CRPBI, FES-Y and PMON) and the subfactors were then loaded onto a main Child Factor. Given the ordinal scales, each item was labeled as ‘categorical’ and all factor variances were constrained to 1. While there are extensive discussions regarding appropriate fit criteria for Confirmatory Factor Analysis (CFA) models (McNeish and Wolf, 2021), here we use thresholds that are comparable to those in the original paper (Thijssen et al., 2020, p. 689). For the Child Factor, in the current sample the fit criteria are reasonable: *χ2*(149) = 1092.6, *p* < .001; Comparative Fit Index (CFI) = 0.97; Tucker-Lewis Index (TLI) = 0.97; Root Mean Square Error of Approximate (RMSEA) = 0.03; Standardized Root Mean Square Residual (SRMR) = 0.05. The loadings of each subscale on the Child Factor are 0.82 for CRPBI, −0.54 for FES-Y and 0.77 for PMON, such that higher scores on the Child Factor reflected *positive* aspects of the environment. Factor scores were extracted and used in subsequent analyses.

#### 2.4.2. Parent items/factor

Like the FES-Y, the parent-reported *Family Environment Scale* (9-item FES-P) is a measure that assesses family social environment as perceived by the family member. Items are averaged and reverse coded such that *higher scores indicate higher perceptions of family conflict* (i.e., higher scores interpreted as a more *positive* family environment). One item measuring conflict from the *Kiddie Schedule for Affective Disorders and Schizophrenia* (KSADS; Kaufman et al., 1997) was used to assess parental-child conflict. *High scores on this item indicate a more negative relationship between the child and parent* (i.e., higher scores interpreted as a more *negative* family environment).

For the current study, the Parent Factor is composed of the individual items for FES-P and KSADS scales. To maintain the item/factor structure from Thijssen et al. (2020), two items from the FES-P (Q7 & Q9) were excluded. Like the Child Factor, a CFA was submitted to *lavaan::cfa()* in R with each measure’s items loading onto the Parent Factor. Given the ordinal scales, each item was labeled as ‘categorical’. Using the fit criteria from the original paper, in the current sample the fit criteria for this factor are reasonable, *χ2*(20) = 947.6, p < .001, CFI = 0.93, TLI = 0.90, RMSEA = 0.08, SRMR = 0.10. For ease of interpretation, we inverted our Parent Factor scores to ensure its consistency with the Family Environment scores (described below). Thus, higher scores on the reverse-coded Parent Factor indicate more *positive* aspects of the environment. Factor scores were extracted and used in subsequent analyses.

#### 2.4.3. Demographic items/factor

Youth self-reported their age in months, their sex at birth using options “Male”, “Female”, “Other”, and race/ethnicity, (1) “White, (2) “Black”, (3) “Hispanic”, (4) “Asian”, (5) “Other”. These variables were used as covariates in the mediation models, consistent with Thijssen et al. (2020).

Parents self-reported on income, education, separation and pregnancy variables. For combined household income, parents selected an income category for the past 12 months ranging from (1) “less than $5000” to (10) “$200,000 and greater”. Parents reported on their or their partner’s highest level of education by selecting an education category that ranged from (0) “Never Attended” to (21) “Doctoral Degree”. Parents reported on their marital status, such as (1) “Married” or (6) “Living with partner” and whether their pregnancy with the child was a planned pregnancy (Yes/No).

Parental psychopathology was assessed using the comprehensive measure from the Achenbach System of Empirically Based Assessment Adult Self-Report (ASRS; Achenbach and Rescorla, 2003). Here, the t-scored total problems score is used (range 25–100), whereby *higher values indicate higher problems*.

For the current study, the Demographic Factor is composed of income, education, separation, pregnancy and the parental psychopathology variables. Like the Parent and Child Factors, confirmatory factor analysis was submitted to *lavaan::cfa()* in R with each measure’s items loading onto the Demographic Factor. Given the ordinal scales, each item except for parental psychopathology was labeled as ‘categorical’. Using the fit criteria from the original paper, in the current sample the fit criteria for this factor are reasonable, *χ2*(5) = 195.4, p < .001, CFI = 0.98, TLI = 0.96, RMSEA = 0.08, SRMR = 0.05. For ease of interpretation, we inverted our Demographic Factor scores to ensure it is consistent with the Family Environment scores (described next). Higher scores on the Demographic Factor indicate more *advantaged* aspects of the environment. Factor scores were extracted and used in subsequent analyses.

#### 2.4.4. Family environment factor

Like the original paper, the Family Environment higher order factor is a confirmatory factor that is composed of the Child, Parent and Demographic variables. The confirmatory factor analysis was submitted to *lavaan::cfa()* in R. In a single model, the youth reported items representing the family environment (i.e., CRPBI, FES-Y, PMON) loaded onto a Child subfactor, the Parent reported items representing the family environment (i.e., FES-P and KSADS) loaded onto the Parent subfactor and the demographic items representing the family environment (i.e., Income, Education, Planned Pregnancy, Parental Separation and Parent ASRS) loaded onto the Demographic subfactor. All subfactors were then loaded onto the overarching Family Environment Factor. All factor variances were constrained to 1. In the model, excluding parental income and parental psychopathology, all categorical items were entered as categorical under the ‘ordered’ option in lavaan:cfa. Using the fit criteria from the original paper, the fit criteria for this factor are reasonable for this sample, *χ2*(458) = 7079.2, p < .001, CFI = 0.89, TLI = 0.89, RMSEA = 0.05, SRMR = 0.07. The standardized loadings are Child (Loading: −0.56; 95% CI: −0.59 to −0.54), Parent (Loading: 0.55; 95% CI: 0.53 to 0.58) and Demographic (Loading: 0.47; 95% CI: 0.45 to 0.49). For ease of interpretation, we inverted our family factor scores to ensure it is consistent with the previous study. Thus, higher scores on the Family Environment Factor indicate a *less stressful* family environment. Factor scores were extracted and used in subsequent analyses.

### 2.5. Pubertal stage

The *Pubertal Development Scale* (PDS; Petersen et al., 1988) assesses the child’s pubertal stage. The PDS is a non-invasive measure that assesses current pubertal status in females and males. Higher scores indicate further progression in puberty. Here we use the (1) youth self-reported average scores to assess youth reported pubertal development, (2) parent reported average scores that assess parent reported pubertal development and (3) as used in prior ABCD studies (Petrican et al., 2021) the average of parent and youth reported average pubertal development scores.

### 2.6. Analytic plan

**Fig. 1.**
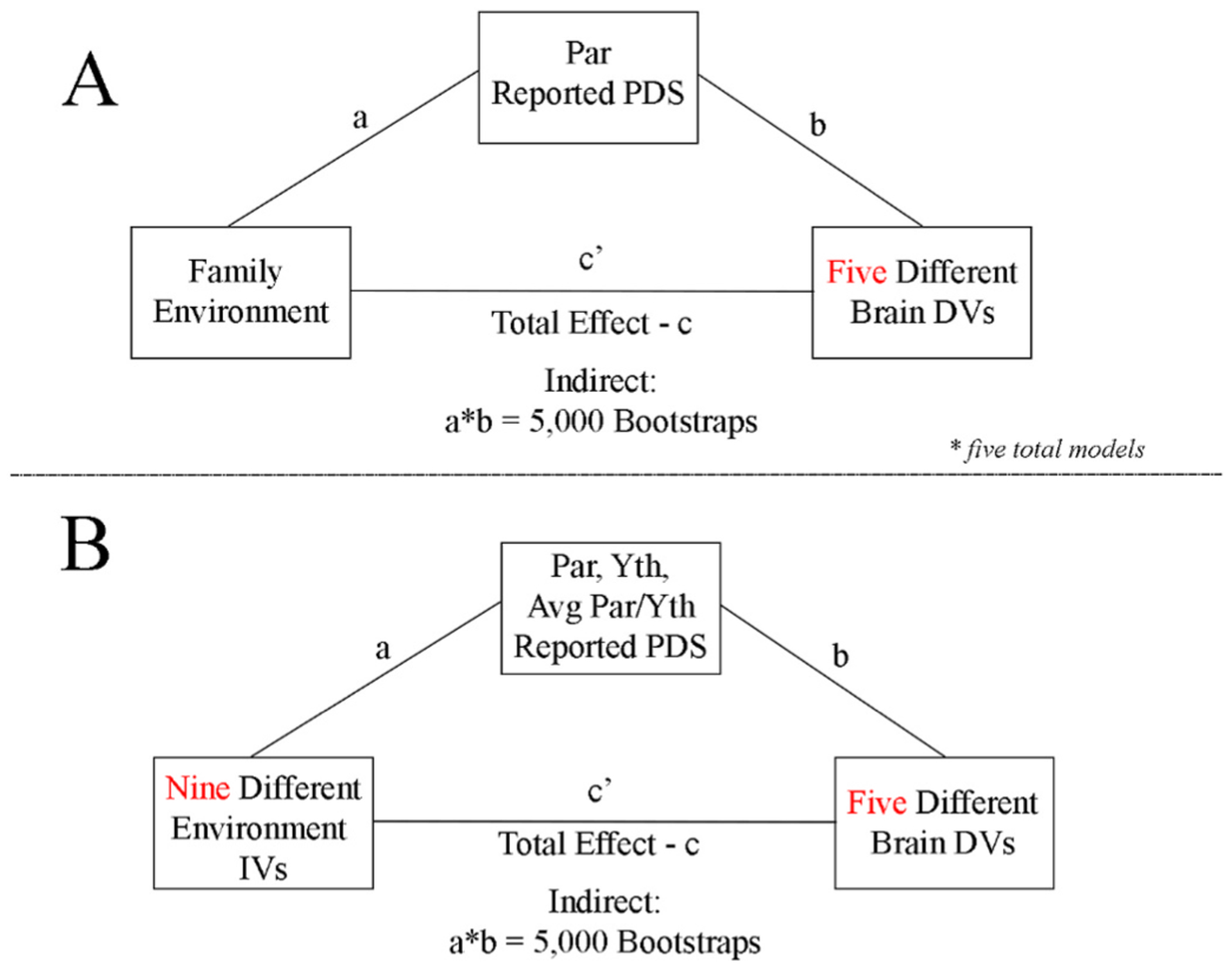
Conceptual model for proposed analyses in Aim 1 and Aim 2. **A**: Mediation model from Thijssen et al. (2020) that is used in replication. **B**: Proposed models for the mediation analyses, varying across nine independent variables (IV) and three mediators.

Descriptive statistics were calculated for key demographic variables for this study. Bivariate Pearson correlations (*r*) are provided for the relations between variables of interest, the subfactors and overarching factors. Distribution plots for each variable are also provided to represent the normality of these variables. Descriptive statistics and the Pearson correlation tables among the demographic and factor variables were provided as part of the pre-registration as they are not central to the conceptual replication (Aim 1) and multiverse analyses (Aim 2); see conceptual Fig. 1.

The core analyses in our pre-registration (https://doi.org/10.17605/OSF.IO/GXK96) were to test the indirect effect of reported pubertal development on the association between the environment and the structure and function of the brain (Thijssen et al., 2020). The mediation model is composed of several parts: path-c, path-a, path-b, path-c’ and the indirect effect, as illustrated in traditional mediation analyses (Baron and Kenny, 1986; Mackinnon and Dwyer, 1993), shown in Fig. 1 and expanded on in the supplemental materials.

In Aim 1, we replicate the core Mplus mediation results from the Thijssen et al. (2020) study using structural equation modeling using *lavaan* (Rosseel, 2012) in R version 4.0.3 (R Core Team, 2020). We re-analyze mediation analyses using the IV (Family Environment Factor), the mediator (parent self-reported pubertal development), and the five brain DVs ((1) Bilateral Amygdala Subcortical (Amygdala volume), (2) Bilateral ACC Cortical Thickness (ACC CT), (3) Bilateral ACC Cortical Area (ACC CA), (4) Cingulo-Opercular Network and Left Amygdala functional connectivity (Left Amyg-CON) and (5) Cingulo-Opercular Network and Right Amygdala (Right Amyg-CON) functional connectivity, see conceptual Fig. 1A). The replication of estimates, specifically indirect and direct coefficients, from the original study and replication in Aim 1 are evaluated using three metrics. First, similar to reports in Open Science Collaboration (2015), for each of the five mediation models in Aim 1, the consistency in a) direction and b) significance of the indirect and direct effects in these analyses and the original published study. Second, acknowledging the sample and measure variability that contribute to our confidence in effects, for each of the five mediation models, we plot the overlap between the a-path, b-path, indirect, direct and total effects from the original study with their 95% confidence interval (CI) for this replication study. Then, similar to reports of replication in Open Science Collaboration (2015), we also do the inverse, whereby we overlap the estimate from the replication with the original studies 95% CI. For the original work, we estimate the upper bound 95% CI by adding 1.96*Standard Error to β and the lower bound 95% CI by subtracting 1.96*Standard Error from β that are published in Figure 1 and Table 4–6 in the original paper (Thijssen et al., 2020). In cases where we could not calculate this from the original paper, such as for indirect and total effects, we received standard error terms from the original study’s authors. Third, out of the list of eight authors on the pre-registered project (https://doi.org/10.17605/OSF.IO/GXK96) that were not part of the original study (Thijssen et al., 2020), MID, DPK, KII, SL and HB were selected to report whether the effect (1) did or did not (0) replicate. Agreement was operationalized as when 80% (4 out of 5) of the authors determined that an effect did or did not replicate. The authors inspected the sample size, beta estimates, 95% CI and *p*-values from both the original and replication study to derive their conclusion. Based on these ratings, we calculate and report the fraction of authors, e.g. n/5, that concluded whether each effect was replicated from the original to the current study. Thus, in the replication we evaluate whether the inference and conclusions from the model are consistent between the studies.

In Aim 2, we extend the mediation results in Aim 1 by evaluating the effects across the theoretically plausible multiverse of the *independent* family environment variables and *mediating* pubertal variables (See Table 1 and Fig. 1B). Specifically, we consider the theoretically plausible independent variables: Child Factor, Parent Factor, Demographic Factor and frequently used scales measuring parental acceptance, parental monitoring and family conflict, as reported by the youth and parents and, given their large correlation (*r* = 0.62), the z-scored average of parent reported income and education. Given the nuance in the pubertal scale and dissimilarity discussed above, we consider the theoretically plausible mediator of youth self-reported, parent reported and the average of youth/parental reported pubertal development stage.

Similar to the multiverse mediation analyses in Rijnhart et al. (2021), in Aim 2, the mediation results across our 135 mediation model permutations are reported using specification curves (Simonsohn et al., 2020). A specification curve is reported independently for the direct, indirect and total effect. This is used to represent the range of estimated effects across the variable permutations. This is reported in two panels. Figure Panel A represents the ordered estimated beta coefficients and their associated significance (null hypothesis is 0) colored based on no significance (gray), negative (red) or positive (blue) significance. Figure Panel B represents the analytic decisions (i.e., IV, DV and mediator) that are in the model that produced these ordered estimates. To draw inferences across the specification curve, we report several results. First, like Rijnhart et al. (2021), we report the frequency and direction of the effect across the multiverse compared to the effects in Aim 1. Second, we consider the proportion of effects from Aim 1 that overlap with the 95% CI in Aim 2 for each brain outcome. Finally, we consider the proportion of values that are significant in the direction that is consistent with Aim 1 results. The latter is solely for reporting the percentage of effects that may go unnoticed given the traditional null-hypothesis testing framework and the *p* < .05 threshold that is often used in psychology research. We set the alpha cut-off (p < .05) for the mediation analyses. This is consistent with recent perspectives on multiple comparison corrections in exploratory work (Rubin, 2021; Thompson et al., 2020). To provide context for deviations across our models, we consider within/between category variation. For instance, we may observe greater similarity in effects across the overarching Family Environment and Parent, Demographics and Child subfactors than the Family Environment factor and individual scales, as the factors may capture more signal and less noise (Hodson, 2021). We report this in a two-panel figure to easily digest the difference in direction and magnitude of effects across factor derived and individual measure scores.

## 3. Results

Characteristics of the sample analyzed in the current study are presented in Table 2. The final sample was composed of 6658 participants (50.6% male). The average age of the participants included in the sample was 119 months, or approximately 10 years old. Parent-reported and child self-reported PDS mean scores were 1.78 and 2.09, respectively. This assessment of pubertal development indicates that participants were, on average, in the “early puberty” stage of pubertal development. Notably, 3.9% of the parent reported and 19.7% of the child reported PDS scores were missing.

**Fig. 2.**
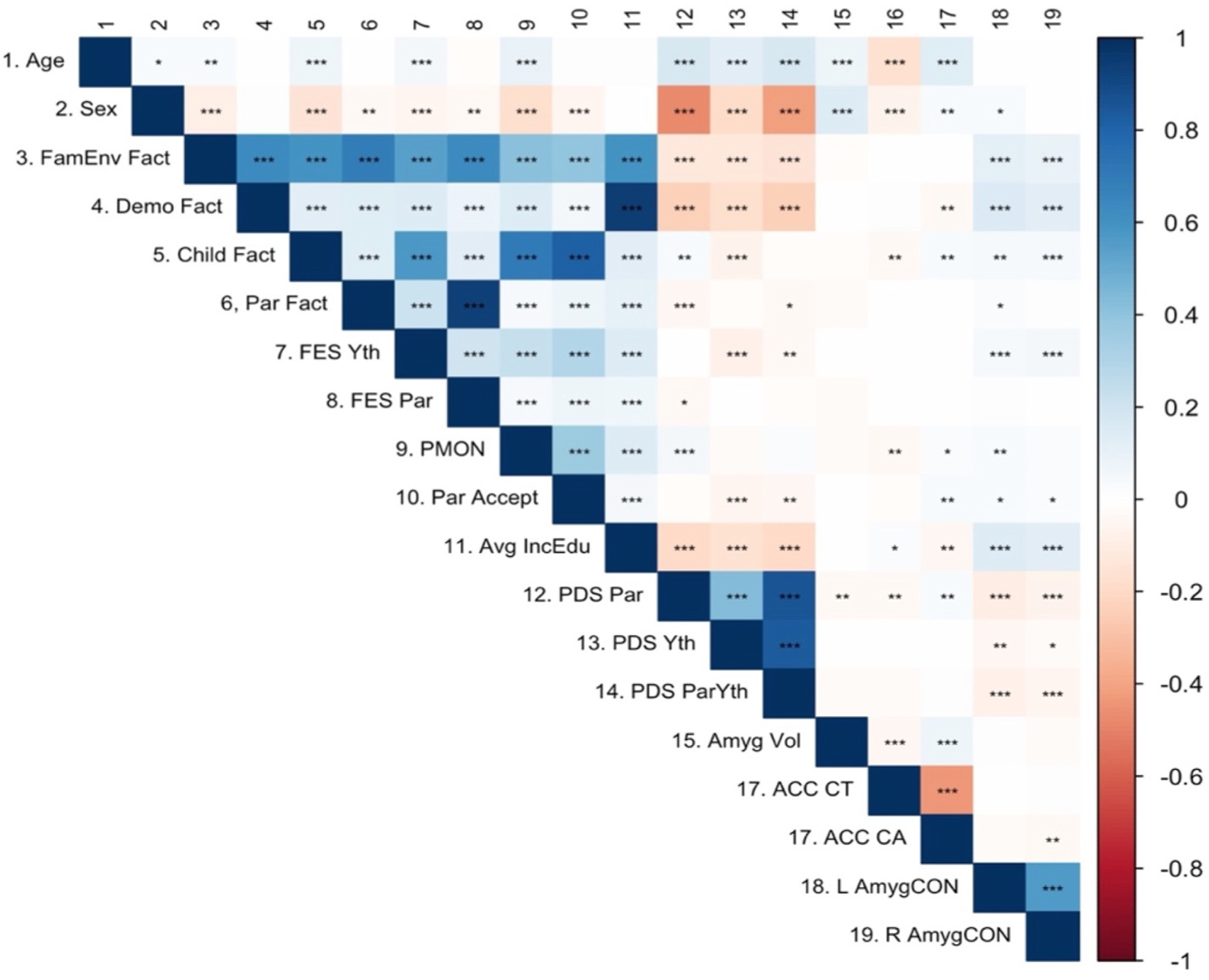
Correlations (*r*’s) between several key variables and factors proposed in Aim 2. Blue-shaded boxes represent positive correlations; red-shaded boxes represent negative correlations; the darkness of the hue represents the magnitude of the correlations. FamEnv = Family Environment; Demo = Demographic; Fact = Factor; Par = Parent; Yth = Youth; FES = Family Environment Scale (i.e. Conflict; reverse scored); PMON = Parental Monitoring; Accept = Child Report of Parent Behavior Inventory (i.e., Acceptance); Avg IncEdu = Average Parent Reported Income & Education; PDS = Pubertal Development Scale; Amyg = Amygdala; ACC = Anterior Cingulate Cortex; CT = Cortical Thickness; CA = Cortical Area; AmygCON = Amygdala Cingulo-Opercular Network connectivity; L = Left; R = Right. For specific *r* point estimates, see Supplementary Table S1. *p < .05, **p < .01, ***p < .001.

Fig. 2 provides the correlations between the environment, puberty and brain measures used in this study (Specific Pearson *r* estimates are available in Supplemental Table S1). As reported in Fig. 2, the demographic factor correlated with parent, child and family environment factors at *r* = 0.13, *r* = 0.13 and *r* = 0.64, respectively. These correlations differed slightly from those in the original study (Thijssen et al., 2020), which reported correlation metrics of *r* = 0.29, *r* = 0.31 and *r* = 0.67, respectively. The correlation among the other factors is comparable to those reported in Thijssen et al. (2020). Specifically, correlations from the replication analyses between the family environment factor and the child factor (*r* = 0.59) and between the family environment factor and the parent factor (*r* = 0.76) are comparable to the original analyses (*r* = 0.70 and *r* = 0.80 for the same analyses, respectively). However, the correlation between parent and child factors (*r* = 0.13) in the replication analyses were different in magnitude than the original analysis (*r* = 0.38).

Then, with the exception of a couple of variables, most of the self-reported variables were weakly correlated (*r* < |0.10|) with the five brain measures. Age was negatively correlated with ACC CT (*r* = −0.17) and positively correlated with ACC CA (*r* = 0.14). Sex was positively correlated with Amygdala volume (*r* = 0.14). The Left Amyg-CON correlated positively with the Family Environment and Demographic Factor scores (*r* = 0.11 & *r* = 0.16) and the averaged income/education (*r* = 0.14). A similar positive correlation was found for the Right Amyg-CON with the Family Environment (*r* = 0.09), Demographic Factor (*r* = 0.12) and the averaged income/education (*r* = 0.12).

### 3.1. Aim 1: Replication of Thijssen et al. (2020)

**Fig. 3.**
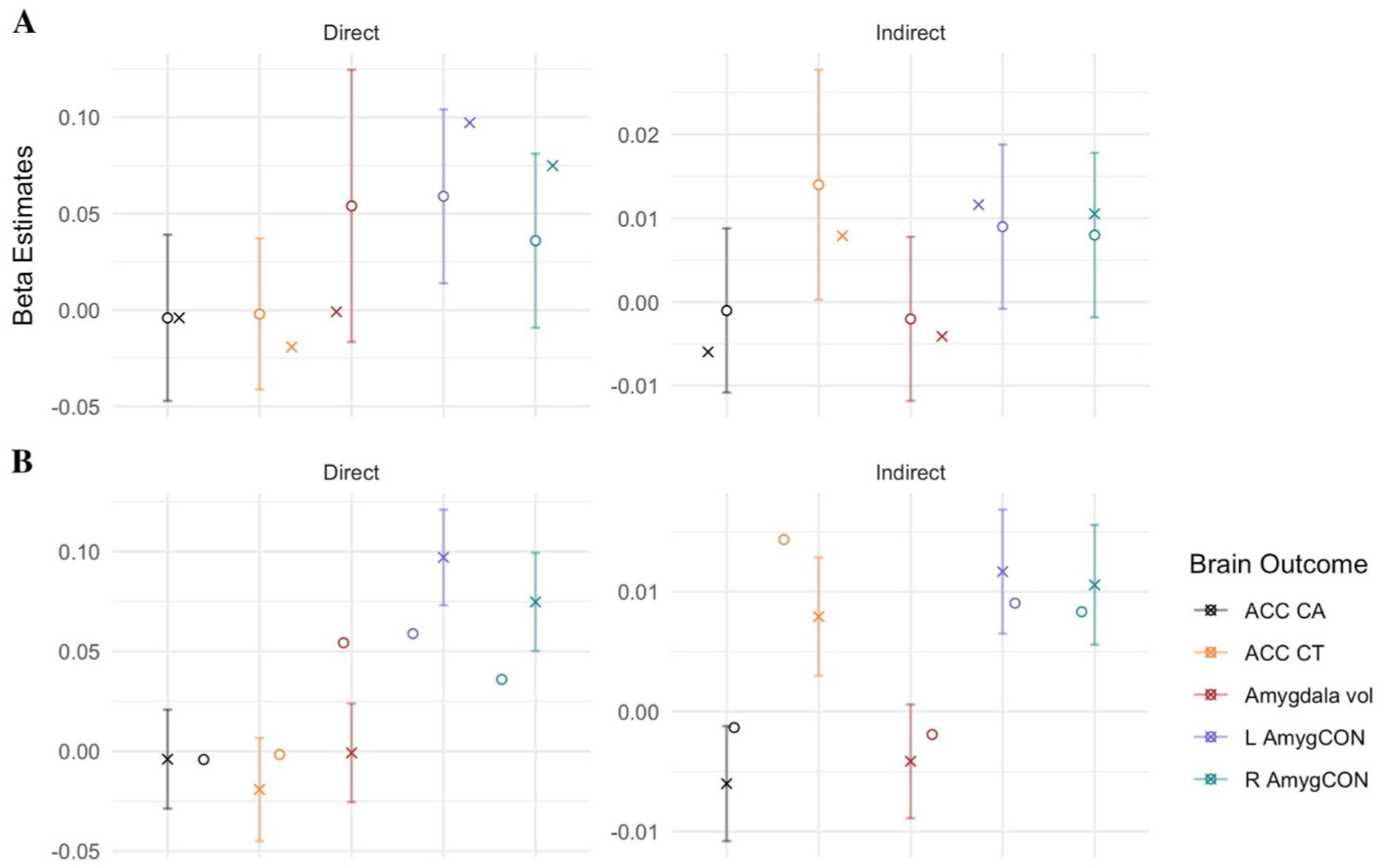
Reported standardized β estimates for Direct and Indirect effects from original study by Thijssen et al. (2020) and Replication study. **A:** Original Study with associated 95% CI = ○; Replication Study = ×. **B:** Original Study = ○; Replication Study with associated 95% CI = ×. ACC = Anterior Cingulate Cortex; CA = Cortical Area; CT = Cortical Thickness; Vol = Volume; L/R Amyg-CON = Left/Right Amygdala Cingulo-Opercular Network connectivity.

The first aim of this study is to evaluate the extent to which the effects reported in Thijssen et al. (2020) using intial ABCD data release replicated in our data using a subsample from the full baseline data. Our preregistration focused on the primary interest of the original work: the mediating role (indirect effect) of parental reported puberty on the association (direct effect) between the family environment and the brain. The two panel Fig. 3 presents the effects from Thijssen et al. (2020; indicated by a circle) and this replication study (indicated by a X). As a reminder, in the original paper, the direct effect was significant for the association between the family environment and the ACC CT and Right Amyg-CON, and the pubertal stage significantly mediated the association between the family environment and the ACC CT. We focused on three metrics for evaluating replication of indirect and direct effects: (1) the direction and p-values of the effects, (2) the overlap in confidence intervals (CIs) and beta estimates between the replication and original analyses and (3) whether most (80%) of raters holistically considered the effect to have replicated.

First, across the five brain regions measured in both the original and replication study, 90% of the estimates (9/10) were in the same direction and 60% of p-values (6/10) were in the same significance category (i.e., *p* > .05 versus *p* < .05) across indirect and direct estimates. Direct effects of family environment on the brain (Right Amyg-CON, *p* < .001) and indirect effects of pubertal stage linking family environment and the brain (ACC CA *p* < .05, Left Amyg-CON, *p* < .001 & Right Amyg-CON *p* < .001) that were significant in the replication study were not significant in the original study.

Second, we considered the overlap between 95% confidence intervals and the reported beta estimates in the original and replication analyses. Here, we considered two options: (a) the extent that the replicated beta effects overlap with the 95% CIs reported in Thijssen et al. (2020) and (b) the extent the beta estimates from Thijssen et al. (2020) overlap with the 95% CI in the replicated models. In the case of the direct and indirect effects, while we found that the beta estimates from the replication models overlapped 100% of the time with the 95% CIs provided in Thijssen et al. (2020) (Fig. 3A), only 50% of the beta estimates from Thijssen et al. (2020) overlapped with the 95% CI found in the replicated models (Fig. 3B). Information across all paths are reported in Supplemental Figs. S3–S4.

Third, five authors rated the extent to which the effects replicated between the two studies. The raters reported using two evaluative techniques, they either (a) interpreted whether the effects and magnitude between the original and replication study are the same or (b) in addition to the direction and magnitude, raters considered whether the difference in *p*-values (e.g., *p* > .05 versus *p* < .05) may impact the takeaway by reader(s) and publisher(s). Across the ten direct and indirect effects for the five key brain areas, raters determined that 60% of the effects replicated, 10% did not replicate and agreement could not be reached on 30% of the effects. Specifically, in terms of indirect effects, the raters deemed that effects replicated for Amygdala volume and ACC CT (100% agreement) and for ACC CA (80% agreement). Less consensus was achieved for Left and Right Amygdala-CON (i.e., 60% agreed that the effects replicated). This is partly due to disagreement among the raters regarding how to compare significance between studies that are differentially powered. For direct effects, the raters agreed that the effects of ACC CT, ACC CA and Left AmygCON replicated (100% agreement), and Amygdala volume replicated (80%% agreement, whereas the effects of Right Amyg-CON did not replicate (i.e., 80% agreement). The completed rates of agreement for each effect are reported in the Supplemental Fig. S2.

### 3.2. Aim 2: Multiverse analyses

The second aim of this study was to evaluate how analytical decisions within a study may influence the results and interpretation of the findings. First, with respect to the independent variable (i.e., different measures of the environment), we considered the similarity and differences across the theoretically plausible subfactors and individual scales. Second, with respect to the mediator (i.e., parent reported, youth reported and youth/parent reported average puberty), we considered the similarity and differences across operationalizations of pubertal development.

**Fig. 4.**
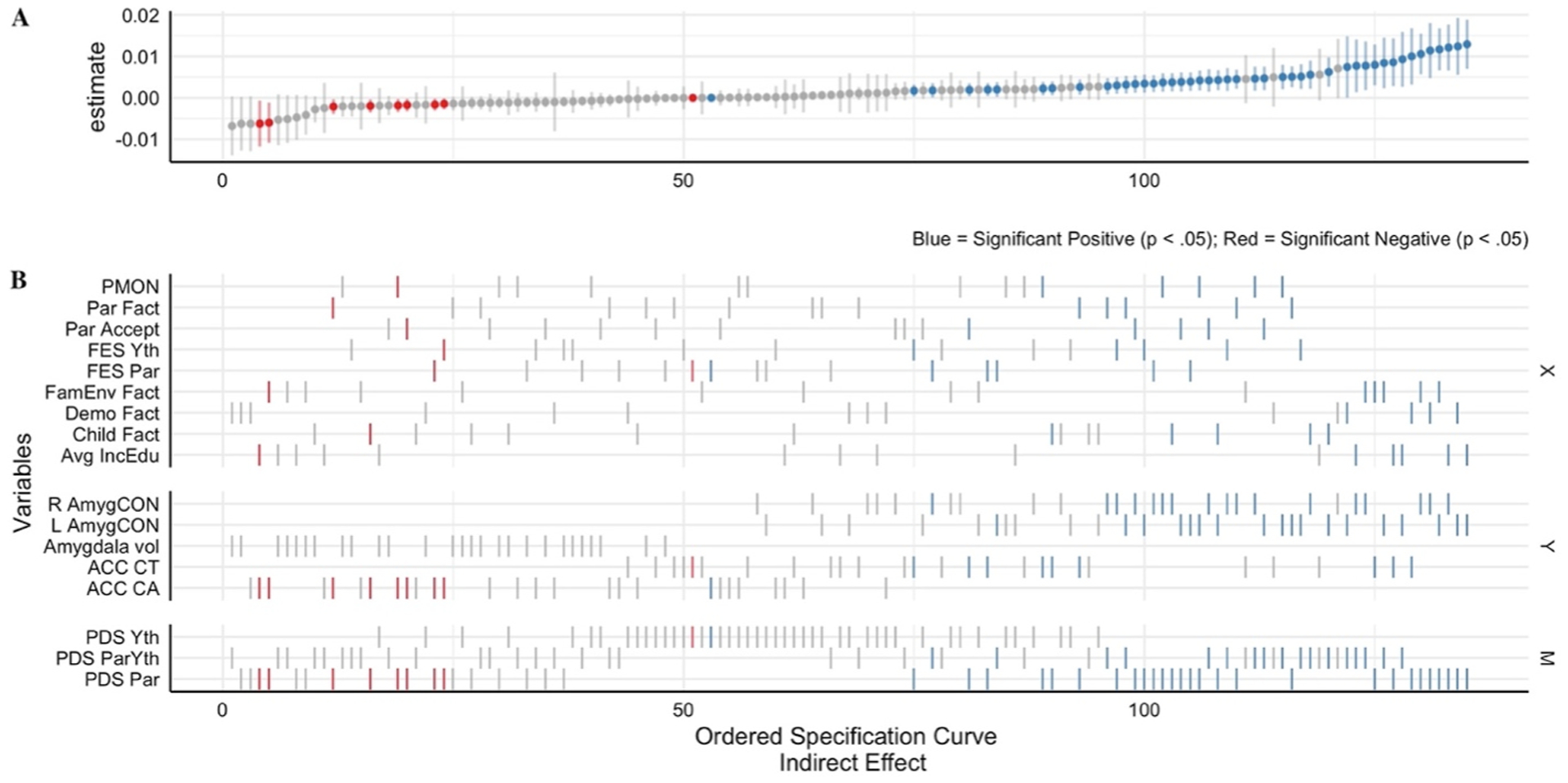
Results of the multiverse analysis expressed as specification curves for all of the 135 models. The blue, gray and red colors indicate whether that standardized β estimate was a significant positive (p < .05), non-significant (p > .05) or a significant negative estimate (p < .05), respectively. Age, sex and race covariates constant across all models. **A**. *Indirect Effect* estimates from mediation models; ordered by size and direction for each estimate for an associated X (predictor), Y (outcome) and M (Mediator). **B**. The associated variables, X, Y and M (Mediator), for each associated effect in the mediation model. PMON = Parental Monitoring; Fact = Factor; Par = Parent; Accept = Child Report of Parent Behavior Inventory (i.e., Acceptance); FES = Family Environment Scale (i.e. conflict; reverse coded); Yth = Youth; FamEnv = Family Environment; Demo = Demographic; Avg IncEdu = Average Parent Reported Income & Education; L/R AmygCON = Left/Right Amygdala Cingulo-Opercular Network connectivity; ACC = Anterior Cingulate Cortex; CT = Cortical Thickness; CA = Cortical Area; PDS = Pubertal Development Scale.

The multiverse analysis for the 135 indirect effects is reported in the specification curve below (Fig. 3). In addition, the specification curve for the direct and total effects is reported as supplemental materials (see Figs. S6 and S7). The specification curve consists of two panels, the estimates (panel A) and variables (panel B). Each estimate in panel A has an associated X (predictor), Y (outcome) and M (mediator) ticked in panel B, which represents the variables used for that mediation model. For example, if we take the 50th estimate reported in panel A of Fig. 4, we observe the estimate is non-significant (gray) with a wide 95% CI that crosses zero. In panel B, we can observe that this same 50th estimate is the mediating (M) role of youth reported puberty on the association between the predictor (X), Youth FES, and the outcome (Y), ACC CT. By cross-referencing the variables for the 50th estimate with the reported estimates from all of the multiverse models (see the supplemental file^1^), we know that the indirect effect for this estimate is β = −0.000013 (95% CI range, 0.003 to −0.003, *p* = .99) and can therefore conclude that selecting this combination of independent variable, mediators and outcome would lead to a non-significant result.

Extending the reported effects in Aim 1, it is therefore plausible to use this approach to investigate the robustness of results when estimating the indirect effect using other predictors and mediators. First, for the Left AmygCON, the three largest significant positive indirect effects in Fig. 4 are the mediating effects of parental reported puberty on the association between the predictor’s average income/education (β = 0.013), demographic factor (β = 0.012) and the family environment factor (β = 0.012) and the outcome Left AmygCON. In a similar model, but for the Right AmygCON and ACC CT, the largest three effects are average income/education (β = 0.012), demographic factor (β = 0.011) and the family environment factor (β = 0.012) for the Right AmygCON, and the demographic factor (β = 0.010), average income/education (β = 0.009) and the family environment factor (β = 0.008) for the ACC CT. Then, the three largest significant positive direct effects for the parent reported pubertal models (see Supplemental Fig. S6) are the direct effect of demographic factor on Left AmygCON (β = 0.134), average income/education on Left AmygCON (β = 0.122) and demographic factor on Right AmygCON (β = 0.108). Finally, across the 135 permutations of the mediation model, 39% of indirect, 42% of direct and 45% of the total effects were significantly different than zero (*p* < .05).

### 3.3. Predictors: Similarity within factors and within scales

**Fig. 5.**
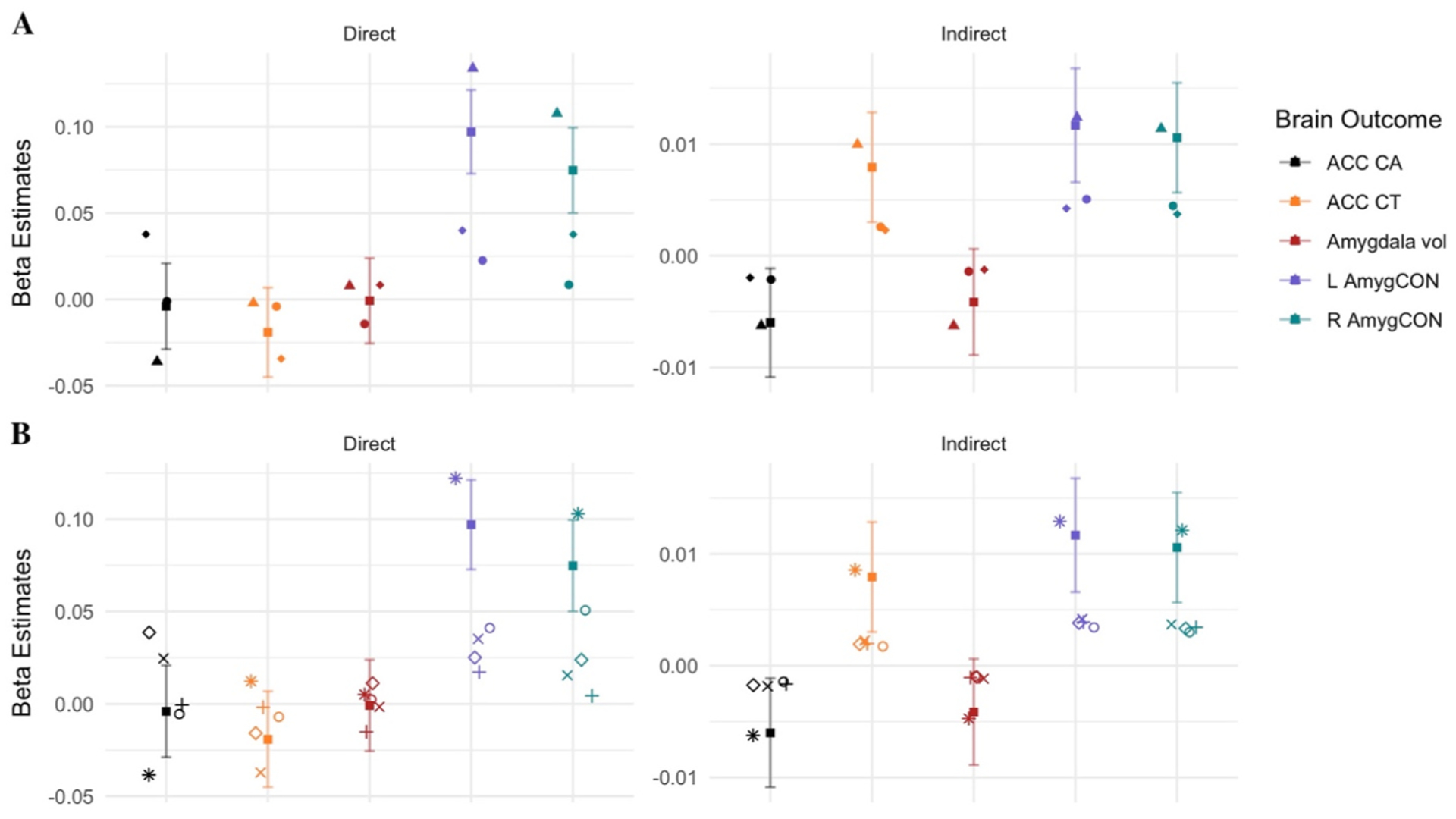
Reported standardized β estimates for direct and indirect effects from the multiverse analyses for Parental Reported Puberty models only. The Family Environment beta and its associated 95% CI is reported in both Panel A & Panel B to provide reference for how much effects diverge from the original model across different operationalizations of the independent variable. **A**: estimates for the models using *factor derived* scores as IV (■ = Family Environment; ▴ = Demographic; ● = Parent; ◆ = Child). **B**: estimates for the models using *measure derived* scores as IV (■ = Family Environment; ○ = FES Youth (i.e., Conflict; reverse coded); + = FES Parent (i.e., Conflict; reverse coded); × = Parental Monitoring; ◇ = Child Report of Parent Behavior Inventory (i.e., Acceptance); * = Avg Income/Education).ACC = Anterior Cingulate Cortex; CA = Cortical Area; CT = Cortical Thickness; Vol = Volume; L/R AmygCON = Left/Right Amygdala Cingulo-Opercular Network Connectivity. See Supplemental Figs. S8–S9 for all paths.

The multiverse analyses also permitted the comparison of the direct and indirect effects between models which include each of the nine different environment independent variables, as shown in panel B of Fig. 1. These nine independent variables can be divided into two categories: factor derived scores (i.e., family environment, parent, demographic and child) and measure derived scores (i.e., FES youth, FES parent, parental monitoring and parental acceptance and average income/education). The estimate for the family environment factor is included in the factor (A) and measure (B) panels in Fig. 5 to provide a reference for the degree to which estimates overlap across the levels of measurement. Panel A of Fig. 5 shows the beta estimates of the direct and indirect effects on the five brain outcomes for each of the factor derived independent variables while Panel B of Fig. 5 shows the same information for each of the measure derived independent variables.

Relative to each other, there was comparatively greater variability in magnitude among beta estimates for the indirect effects, particularly the AmygCON. Regarding the factor derived scores in particular, there were significant results for all four predictors for the ACC CT brain region and both left and right Amyg-CON. Although the magnitude and direction of estimates including these predictors across these brain outcomes are similar, standard errors were lower for parent and child factors compared to family environment and demographics.

### 3.4. Mediator: Similarity across measures of pubertal development stage (PDS)

A primary motivation for the multiverse analysis was to consider how variations among PDS assessment may impact the underlying results. For the direct effects, across the 135 multiverse analyses, we found comparable rates of significance across methods of assessing PDS. Specifically, 42% (19/45) of the parent reported PDS models, 44% (20/45) of the youth reported PDS models and 42% (19/45) of the parent & youth PDS average models had a significant direct effect. In comparison, for the indirect effects, 76% (34/45) of the parent reported PDS models, 0% (0/45) of the youth reported PDS models and 38% (17/45) of the parent & youth PDS average models had a significant indirect effect. Youth reported PDS models had a lower number of significant a-paths (IV → M) than parent and youth/parent averages, 78% (35/45) versus 100%. Notably, youth reported PDS models had a substantially lower number of significant b-paths (M → DV) than parent and youth/parent averages, 4% (2/45) versus 78% (35/45) for parent reported PDS and 40% (18/45) for average parent/youth reported PDS. This might suggest that in the context of analytic flexibility, the construct with which pubertal development is defined significantly impacts the underlying associations and interpretations of the path model.

## 4. Discussion

The current study builds on the analyses of the ABCD data in Thijssen et al. (2020) by conducting a replication and multiverse extension of the original study. In the primary mediation analyses, Thijssen et al. (2020) reported a significant direct effect between the overarching family environment factor and ACC CT and Right Amyg-CON, and a significant indicted effect, whereby parent reported pubertal development mediated the association between the overarching family environment factor and ACC CT. Our study provides a direct replication and extension of an existing study using the publicly available ABCD data. We replicated a subset of the direct effects for the association between the family environment and five brain regions and the indirect effect of parent reported pubertal development from Thijssen et al. (2020), which suggests some behavior-brain associations may change between the releases of the ABCD data. In the multiverse analyses, we found that, across the four factor-derived scores and five individual measures, there was a higher degree of variability in the magnitude and direction among beta estimates for the direct effects than the indirect effects, which suggests there are nuanced differences in effects depending on how the environmental variable is operationalized. In the case of the indirect effects, results were most consistent for the family environment, demographic and income/education independent variables and the parent reported pubertal measures, which suggests a substantial amount of shared variance among variables representing SES. Collectively, these findings demonstrate the importance and necessity of clarifying whether effects replicate, whether nuanced differences in operationalizations of the family environment impact conclusions about neurodevelopment and the potential consequences of using different pubertal scales on the underlying results.

We used three metrics to compare the results of the original and direct replication analyses which yielded somewhat different information. The three metrics we used were: 1) the consistency in direction and significance of effects, 2) whether the 95% confidence intervals from the original and replicated studies included the effects from the other study and 3) subjective ratings from the authors. First, while 90% of the estimates in the current study were in the same direction, 60% of *p*-values were in the same category (e.g., *p* > .05, *p* < .05) as in the original study. Specifically, several direct and indirect effects that were not significant in the original work were significant in the replication. Statistically, this can, in part, be attributable to the difference in the sample size (Wagenmakers, 2007) and negligible correlations becoming significant with increased power (Cohen, 1994), as the replication sample was over two times larger than the original study, which yielded greater evidence against the null hypothesis. The significance threshold (e.g., *p* < .05) is traditionally used to evaluate whether a finding or set of findings is published or promoted versus ending in the file drawer (Simonsohn et al., 2014). This threshold indicates that an estimate is significantly different from zero but it doesn’t necessarily indicate whether two estimates significantly differ (Gelman and Stern, 2006). Second, it is notable that the original study reported wider confidence intervals compared to our replication study. This, in part, explains why only 50% of the beta estimates from the original study overlapped with the 95% CIs in this replication study, whereas all the beta estimates from the replication study overlapped with the 95% CIs provided in the original work. This difference suggests that the model estimates for the effects reported in the original study had greater uncertainty around the estimated betas than those reported in the replication given the larger sample size of the replication. Third, across subjective ratings, the raters agreed that 60% of the effects replicated, 10% did not replicate and 30% could not achieve consensus among the majority of the raters. For example, consensus was not reached for mediating effect of parent reported puberty between the overarching family environment factor and Left and Right Amyg-CON functional connectivity. This is, in part, reflects the two distinct ways raters judged the replication: (1) some raters judge an effect replicated when the effect between the original and replication study were in the same direction and magnitude, irrelevant of the people; (2) other raters judged the magnitude, direction and the *p*-values as the *p*-value may bias interpretation and publishing of a result. This highlights a critical, and still unresolved, discussion of what is meaningfully important when interpreting results between studies and conducting replication efforts (Dick et al., 2021; Romero, 2019).

Replicability is considered to provide a metric of robustness within a scientific discipline through direct or conceptual replications (Romero, 2019; Zwaan et al., 2018) and information about the generalizability of a theoretical framework (Irvine, 2021). The inconsistency among these three metrics reiterate the challenge of quantifying replication and poses a conceptual question: what does it truly mean to *replicate* a study? Fletcher (2021) acknowledges the inherent difficulty in “quantifying” replication. Despite different procedures that exist to establish whether replication has been successful, Zwaan and colleagues acknowledged, “*Two researchers can look at the same replication study and come to completely different conclusions about whether the original effect was successfully duplicated*” (2018, p. 12). Thus, defining a consistent conceptual definition of replication remains an ongoing challenge. For instance, consider a situation in which the direction and significance of an effect are replicated, but the magnitude of the effect differs considerably from the original study. Would one argue that the original study has not been replicated? Would policymakers act on the findings from the original and the replicated study in the same way? In the current replication study, we have determined that a portion of findings from the original study were replicated based on the three metrics used. However, additional consideration should be given to the conceptual meaning of replication while interpreting the current magnitude and direction of the current effects.

The changes in the magnitude of effects during a replication are especially important in the context of multiple comparisons. In our replication we compared uncorrected significance values with the uncorrected values in Thijssen et al. (2020). Recently, using the second release of the ABCD study data, the original team conducted a replication of their study (Thijssen et al., 2020) and found some of their conclusions changed (Thijssen et al., 2022). Specifically, they reported that in the new data the mediating effect of parent reported pubertal development on the association between family environment and ACC CT and Left Amyg-CON were significant. However, the effect for the ACC CT was no longer significant using multiple comparison correction. Yet, using a conservative Bonferroni correction (α/5 models), the ACC CT model would remain significant in our replication study. Highlighting how differences in sample size and multiple comparison corrections can alter conclusions and make conclusions about replication even more challenging.

Results from the multiverse analysis in the present study also demonstrated the important role of variable selection in determining magnitude and direction of effect sizes. Here, we found that the association between family environment and specific neural structures and functional connectivity can differ by how the family environment was operationalized. While we found consistent findings with the original paper that parent-reported pubertal development indirectly mediated the association between family environment factor and ACC CT (Thijssen et al., 2020), we also found equally sized effects using other socioeconomic measures, such as a demographic factor and parent reported income/education. It is reasonable to speculate that these findings were driven by the fact that these variables are highly correlated and thus play a similar role in the association with brain and pubertal development (Conger et al., 2010; Linver et al., 2002); however, these findings also illustrate that differences in the effects of the environment on brain and development could be explained by differential approaches in statistical modeling, namely the use of latent variables (Mersky et al., 2017; Smith and Pollak, 2020). While capturing shared variance among multiple variables using latent factors may provide a more overarching representation of family environments, latent factors may also conceal important specificity that exists in each individual component. Thus, our multiverse approach provides a model where both the overarching and individual components can be accomplished by reporting all possible permutations of variable selection.

In the context of operationalizing a variable, one of the constructs in the adolescent literature that shows the most notable variation in operationalization is puberty. In particular, whether the most accurate and reliable judgments of it come from children themselves or from their parents. While the majority of the ABCD literature uses the parent reported PDS (Demidenko et al., 2021; McNeilly et al., 2021; Thijssen et al., 2020), there are also instances of the youth reported PDS (Argabright et al., 2022) and an average of the parent and youth reported PDS being used (Petrican et al., 2021). This multiverse study provided a unique opportunity to compare the consequences of researchers utilizing each of these three operationalizations of puberty. In these analyses, the direct effect between the environmental predictors and the brain outcomes was minimally affected by how puberty was controlled; however, how puberty was operationalized had a substantial impact on the indirect effect. Whereas the indirect effects were significant in 78% of the models that included parent reported PDS, 0% of indirect effects were significant in the child reported models. Although different measures of puberty produced different conclusions, we cannot infer that in any case we had more accurately measured the target of puberty in any of the operationalizations (Irvine, 2021), nor that this conclusively shows that parent reported puberty should be preferentially used, only that how we operationalize puberty might impact discussions about the effects of the environment on brain development. This trend could potentially be dependent on the age of the sample: in these analyses, the ABCD sample was limited to 9- and 10-year-olds, an age group that shows relatively little overt physical manifestations of pubertal development by this point, particularly in boys. In addition, a potential concern is the relatively high amount of missing data for the youth-reported PDS (19.7%) compared to the parent-reported PDS (3.9%) in the final sample, which could also feasibly be directly related to the participants being in the earliest stages of puberty. Pubertal hormone measures acquired through saliva samples were also collected by the ABCD study and could be used as an additional operationalization of puberty, but there are important concerns about the feasibility of using salivary samples, namely the representativeness of single hormone assays given our knowledge of the extent that hormones fluctuate (Cheng et al., 2021). As the ABCD sample ages, follow-up studies will be needed to make more consistent conclusions about the differences between the different operationalizations of the PDS measures, including biological measures.

It is well understood that measurement error and researcher degrees of freedom contribute to replication (Loken and Gelman, 2017) and generalizability issues (Flake et al., 2022), making multiverse analyses an effective way to meaningfully gauge the robustness of findings. There is increasing evidence from the neuroimaging literature that decision points across an analytic pipeline may impact the conclusions when reproducing or replicating brain-behavior effects (Bloom et al., 2021; Botvinik-Nezer et al., 2020; Bowring et al., 2022; Bryce et al., 2021; Li et al., 2021). These issues are confounded in developmental research on environmental stress as a product of overlapping measures and competing theories (Smith and Pollak, 2020). The nature of economic interests (Mischel, 2008; Romero, 2019), high rate of psychologists self-reporting engagement in questionable research practices (John et al., 2012), pressing need for publishing significant (Romero, 2019; Zwaan et al., 2018) and novel findings (Proulx and Morey, 2021) and ‘hindsight bias’ (Klapwijk et al., 2021; Zwaan et al., 2018) makes it challenging to decipher which specific researcher decisions would reproduce estimates in the data.

So, what can we learn from a multiverse analysis? Given competing theoretical frameworks in the environmental stress literature, defining what is an ‘ambiguous’ decision in the analytic process is challenging. Nevertheless, multiverse analyses offer researchers a suitable approach to probe the sensitivity, or robustness, of their results. Multiverse analyses are not intended to disprove an overarching model, instead, they offer awareness of alternative explanations that are plausible given the shared nomological space of the measured variables. This approach allows researchers the opportunity to empirically examine competing models that are often influenced by researchers’ degrees of freedom and theoretical perspectives (Smith and Pollak, 2020). For example, compared to an overarching variable (such as the family environment), in the context of pubertal development a simplified version of the independent variable (such as income and education) may be comparatively more interpretable for intervention researchers. By providing alternative results with a multiverse analysis, the intention is to confer improved communication of results to permit researchers to evaluate under which conditions the alternative explanations could also be true. When studying environmental stress, applying multiverse analyses is especially relevant given that measures of adversities overlap in a number of ways and therefore are difficult to tease apart in correlational work (Smith and Pollak, 2020).

### 4.1. Study considerations

This study contains several notable considerations which should be taken into account when interpreting the replication and multiverse analyses.

First, while we use comparable metrics to prior replication studies (Open Science Collaboration, 2015), some researchers may disagree with our definitions of replicated and non-replicated effects. Fletcher notes the limitation of the dichotomous nature of null hypothesis significance testing (NHST), in that the ‘*the facts of replication are objectively conventional*’ (2021, p. 56). Although basing judgments concerning replication within a NHST framework could be considered a limitation, here we consider this to be a valuable metric as researchers and clinicians may reach different conclusions based on estimates that are and are not significant. Given that the 95% CI and *p*-value are susceptible to similar critiques, we addressed this potential limitation by using a collection of values and statistics that were then critically assessed by a random subset of coauthors. Although Fletcher (2021) highlights that subjective assessments, too, may be unreliable indicators, human judgment is deeply embedded throughout the extant literature, from the processes of deciding which theory to base a hypothesis on to which scientific evidence provides support for results from a given analysis. Nevertheless, we agree that there are biasing factors in using a subjective assessment of replication and a Bayesian approach, such as replication Bayes factors (Ly et al., 2019), should preferably be considered for future replication and extension analyses, if both the original and replication data are easily accessible.

Second, future studies could improve on the procedure for the subjective assessment of replication. While we were intentional in ensuring that the assessors were independent of the original study’s co-author list, there could potentially be greater confidence in the conclusion of the subjective assessment with a greater number of assessors (this study used a report of five co-authors). Similar to how neuroimaging studies source broad teams to redo analyses (Botvinik-Nezer et al., 2020), crowdsourcing methods may be a reasonable approach to increase the number of subjective assessments, provided that assessors were provided with enough information and independence from the research teams.

Third, some of the effects in this study and the original study (Thijssen et al., 2020) could potentially be considered as small in magnitude and pose important considerations. Notably, small effects in fMRI brain wide associations for developmental phenotypes (Marek et al., 2022) and the increasing understanding that small effects in large data may be commonplace (Dick et al., 2021). Hence, there is a strong argument for adjusting expectations regarding the magnitude of effects from large, well-powered datasets, such as the ABCD data (Owens et al., 2021). This is confounded with large datasets, such as the ABCD and UK Biobank studies, which are adequately powered to identify significant correlations that are likely negligible (Cohen, 1994). Given this, the variability around estimates and decreased sample sizes in the partial release would be affected based on whether conclusions reached significance using traditional (*p* ≤ .05; Wagenmakers, 2007) and newer significance thresholds (*p* ≤ .005; Lakens et al., 2018). In future replications, instead of setting α cutoffs for significance, researchers should supply bounds within which an estimate is deemed replicated which is irrespective of the *p*-value.

Fourth, while not central to this study but critical to takeaways from the multiverse analyses, a key consideration for our use of pubertal scales is the causal nature of the variables in our meditation models (Rohrer et al., 2021). Generally, like the environmental variables, the child and parent reported pubertal developmental scales are self-reported items that may incur some biases. In the conceptual paths, several linear relationships are assumed to exist based on detailed theoretical frameworks in Thijssen et al. (2020). Nevertheless, adolescent pubertal development “*acts on re-activation of specific neuroendocrine systems*” (Forbes and Dahl, 2010, p. 67); therefore, knowing the temporal nature of environmental stressors may be important to understand its effects on neural and pubertal development. While some effects may be small in a cross-sectional analysis, the cumulative associations among the environment, puberty and brain may be substantial over the course of development (Funder and Ozer, 2019). In the context of the replication and multiverse analyses, the causal and cumulative effects cannot be accounted for in these data.

Finally, the analyses here focused solely on the tabulated brain imaging data provided by the ABCD consortium and did not focus on alternative operationalizations of brain data. Based on recent analyses demonstrating the impact of decisions within an analytic pipeline (Bloom et al., 2021), between analytic pipelines in neuroimaging (Li et al., 2021) and different brain parcellations on the resulting estimates (Bryce et al., 2021), this multiverse analysis was not well positioned to answer questions regarding how differences in the operationalization of brain variables impacted the mediating models. Future studies focused on replications and multiverse analyses of ABCD data should consider using a combination of data that are tabulated by the consortium and those that test deviations from those methods. This is especially important as the consortium makes updates and/or changes to the preprocessing pipelines.

## 5. Conclusion

The future of developmental science will increasingly involve large consortium secondary datasets, such as the ABCD study, to answer valuable developmental questions. Evaluating how effects replicate between teams of researchers, across releases and with different variable permutations will be an important part of the process to ensure the robustness of findings. We conducted a replication and extension of a previously published study using measures of environmental experiences, pubertal development and brain structural and functional variables. Specifically, we evaluated the convergence between conclusions from the original and replicated study and across alternative operationalizations of the environment and pubertal development variables. Despite the similarities in the study design, based on the assessment of effects and statistical parameters, only a portion of effects were deemed to have replicated. In the case of the multiverse analysis, we found the mechanistic role of puberty in the association between the environment and the brain may be, in part, impacted by how the environment is operationalized, but was consistently altered by how puberty is operationalized in the data. This study demonstrates the nuance across environmental variables in the ABCD data and lack of consensus across parent and child reported puberty scales.

## Supplementary Material

1

2

## Figures and Tables

**Table 1:** Aim 2 variables for multiverse analyses: IV*M*DV = 135 total mediation models.

Environment (IV)	Puberty Scale (M)	Brain (DV)	Covariates (constant)
(1) Family Environment Factor	(1) Parent Reported PDS	(1) Bilat. Amygdala SV	Age, Race/Ethnicity, Sex
(2) Parent Factor	(2) Youth Reported PDS	(2) Bilat. ACC CT	
(3) Child Factor	(3) Averaged Parent/Youth PDS	(3) Bilat. ACC CA	
(4) Demographic Factor		(4) Left Amyg-CON rsfMRI	
(5) FES Youth		(5) Right Amyg-CON rsfMRI	
(6) FES Parent			
(7) Parental Monitoring			
(8) Parental Acceptance			
(9) z-scored Parental Income/Education			

PDS = Pubertal Development Scale; FES = Family Environment Scale (reverse scored); Bilat = Bilateral Average; ACC = Anterior Cingulate Cortex (rostral/caudal average); Amyg-CON = Amygdala Cingulo-Opercular Network Connectivity; SV = Subcortical Volume; CT = Cortical Thickness; CA = Cortical Area; IV = Independent Variable; M = Mediator; DV = Dependent Variable.

**Table 2:** Sample descriptives across different releases and for final sample.

	Total Release	Release Pre Aug 8, 2018	Release Post Aug 8, 2018	Final Sample
	N = 11,878	N = 4743	N = 7135	N = 6658
	*Mean (SD)*			
**Age (Months)**	119 (7.50)	120 (7.33)	118 (7.53)	119 (7.46)
**Parent Report - PDS**	1.76 (0.868)	1.72 (0.850)	1.78 (0.880)	1.78 (0.876)
Missing, *N* (%)	472 (4.0%)	136 (2.9%)	336 (4.7%)	261 (3.9%)
**Youth Report - PDS**	2.08 (0.834)	2.08 (0.833)	2.08 (0.834)	2.09 (0.828)
Missing, *N* (%)	2336 (19.7%)	415 (8.7%)	1921 (26.9%)	1312 (19.7%)
	*N* (%)			
**Sex**				
Female	5682 (47.8%)	2262 (47.7%)	3420 (47.9%)	3291 (49.4%)
Male	6196 (52.2%)	2481 (52.3%)	3715 (52.1%)	3367 (50.6%)
**Family Income**				
Less than $5000	417 (3.5%)	104 (2.2%)	313 (4.4%)	253 (3.8%)
$5000 through $11,999	421 (3.5%)	133 (2.8%)	288 (4.0%)	235 (3.5%)
$12,000 through $15,999	274 (2.3%)	79 (1.7%)	195 (2.7%)	155 (2.3%)
$16,000 through $24,999	524 (4.4%)	186 (3.9%)	338 (4.7%)	296 (4.4%)
$25,000 through $34,999	654 (5.5%)	237 (5.0%)	417 (5.8%)	399 (6.0%)
$35,000 through $49,999	934 (7.9%)	363 (7.7%)	571 (8.0%)	519 (7.8%)
$50,000 through $74,999	1499 (12.6%)	634 (13.4%)	865 (12.1%)	820 (12.3%)
$75,000 through $99,999	1572 (13.2%)	692 (14.6%)	880 (12.3%)	906 (13.6%)
$100,000 through $199,999	3315 (27.9%)	1382 (29.1%)	1933 (27.1%)	1800 (27.0%)
$200,000 and greater	1250 (10.5%)	556 (11.7%)	694 (9.7%)	683 (10.3%)
Refuse to answer	512 (4.3%)	192 (4.0%)	320 (4.5%)	289 (4.3%)
Don’t Know	504 (4.2%)	185 (3.9%)	319 (4.5%)	301 (4.5%)
**Education**				
Never attended/Kindergarten only	0 (0%)	0 (0%)	0 (0%)	0 (0%)
1st grade	2 (0.0%)	0 (0%)	2 (0.0%)	1 (0.0%)
2nd grade	1 (0.0%)	1 (0.0%)	0 (0%)	1 (0.0%)
3rd grade	10 (0.1%)	3 (0.1%)	7 (0.1%)	7 (0.1%)
4th grade	8 (0.1%)	1 (0.0%)	7 (0.1%)	5 (0.1%)
5th grade	3 (0.0%)	0 (0%)	3 (0.0%)	2 (0.0%)
6th grade	62 (0.5%)	21 (0.4%)	41 (0.6%)	41 (0.6%)
7th grade	21 (0.2%)	7 (0.1%)	14 (0.2%)	13 (0.2%)
8th grade	61 (0.5%)	22 (0.5%)	39 (0.5%)	36 (0.5%)
9th grade	136 (1.1%)	39 (0.8%)	97 (1.4%)	82 (1.2%)
10th grade	107 (0.9%)	38 (0.8%)	69 (1.0%)	62 (0.9%)
11th grade	193 (1.6%)	56 (1.2%)	137 (1.9%)	118 (1.8%)
12th grade	182 (1.5%)	55 (1.2%)	127 (1.8%)	106 (1.6%)
High school graduate	992 (8.4%)	339 (7.1%)	653 (9.2%)	555 (8.3%)
GED or equivalent	268 (2.3%)	77 (1.6%)	191 (2.7%)	156 (2.3%)
Some college	1950 (16.4%)	753 (15.9%)	1197 (16.8%)	1087 (16.3%)
Associate: Occupation	874 (7.4%)	335 (7.1%)	539 (7.6%)	466 (7.0%)
Associates: Academic	664 (5.6%)	258 (5.4%)	406 (5.7%)	370 (5.6%)
Bachelor’s degree	3333 (28.1%)	1452 (30.6%)	1881 (26.4%)	1849 (27.8%)
Master’s degree	2280 (19.2%)	984 (20.7%)	1296 (18.2%)	1259 (18.9%)
Professional School (MD)	334 (2.8%)	147 (3.1%)	187 (2.6%)	205 (3.1%)
Doctoral degree	380 (3.2%)	149 (3.1%)	231 (3.2%)	228 (3.4%)
Refuse to Answer	17 (0.1%)	6 (0.1%)	11 (0.2%)	9 (0.1%)
**Race**				
White	6182 (52.0%)	2777 (58.5%)	3405 (47.7%)	3427 (51.5%)
Black	1784 (15.0%)	468 (9.9%)	1316 (18.4%)	897 (13.5%)
Hispanic	2411 (20.3%)	934 (19.7%)	1477 (20.7%)	1536 (23.1%)
Asian	252 (2.1%)	106 (2.2%)	146 (2.0%)	148 (2.2%)
Other	1247 (10.5%)	458 (9.7%)	789 (11.1%)	650 (9.8%)
**Parents’ Marital Status**				
Married	7991 (67.3%)	3349 (70.6%)	4642 (65.1%)	4506 (67.7%)
Widowed	97 (0.8%)	49 (1.0%)	48 (0.7%)	40 (0.6%)
Divorced	1082 (9.1%)	470 (9.9%)	612 (8.6%)	567 (8.5%)
Separated	464 (3.9%)	152 (3.2%)	312 (4.4%)	262 (3.9%)
Never Married	1460 (12.3%)	449 (9.5%)	1011 (14.2%)	808 (12.1%)
Living with Partner	688 (5.8%)	253 (5.3%)	435 (6.1%)	411 (6.2%)
Refused to Answer	94 (0.8%)	21 (0.4%)	73 (1.0%)	62 (0.9%)
**Before/After Aug 30, 2017 Cut-off** ^#^				
Release 1	4743 (39.9%)	–	–	2482 (37.3%)
Release 2	7135 (60.1%)	–	–	4176 (62.7%)

# Release 1.1 was released in 2018 and contained part (participant’s data collected before August 30, 2017) of the cohort recruited for the ABCD study. Release 2.0 was released in 2019 and contained the full baseline cohort (participant’s data collected after August 30, 2017) for the ABCD study. Release 3.0 was released in 2020 containing initial longitudinal data. The ABCD Consortium posts information about recent and new releases at the following webpage: https://abcdstudy.org/scientists/data-sharing/.

## Data Availability

As described in the methods section of the manuscript, the data used here are associated with the ABCD Study 3.0 Data Release. Specifically, the NDA #1182451 package (DOI: 10.15154/1519007) was downloaded using the NDA Download Manager and associated data text files were read into R. As described on the ABCD study website (https://abcdstudy.org/scientists/data-sharing/), researchers can access this data by creating an account on the NIMH Data Archive system and requesting access. To ensure the openness and reproducibility of our analyses, all code is publicly available and thoroughly commented in the R files on Github (see Demidenko, 2022).

## Footnotes

1 Supplemental File: MultiverseResults.csv.

## References

[R1] Achenbach TM, Rescorla LA, 2003. Manual for the ASEBA Adult Forms & Profiles. Burlington, VT. University of Vermont, Research Center for Children, Youth, & Families.

[R2] Argabright ST, Visoki E, Moore TM, Ryan DT, DiDomenico GE, Njoroge WFM, Taylor JH, Guloksuz S, Gur RC, Gur RE, Benton TD, Barzilay R, 2022. Association between discrimination stress and suicidality in preadolescent children. Focus 20 (2), 252–262. 10.1176/appi.focus.22020005. PMC10153507 37153135

[R3] Artner R, Verliefde T, Steegen S, Gomes S, Traets F, Tuerlinckx F, Vanpaemel W, 2021. The reproducibility of statistical results in psychological research: an investigation using unpublished raw data. Psychol. Methods 26 (5), 527–546. 10.1037/met0000365. 33180514

[R4] Barch DM, Albaugh MD, Avenevoli S, Chang L, Clark DB, Glantz MD, Hudziak JJ, Jernigan TL, Tapert SF, Yurgelun-Todd D, Alia-Klein N, Potter AS, Paulus MP, Prouty D, Zucker RA, Sher KJ, 2018. Demographic, physical and mental health assessments in the adolescent brain and cognitive development study: rationale and description. Developmental Cognitive Neuroscience 32, 55–66. 10.1016/j.dcn.2017.10.010. 29113758 PMC5934320

[R5] Baron RM, Kenny DA, 1986. The moderator-mediator variable distinction in social psychological research: conceptual, strategic, and statistical considerations. J. Pers. Soc. Psychol 51 (6), 1173–1182. 10.1037//0022-3514.51.6.1173. 3806354

[R6] Belsky J, 2019. Early-life adversity accelerates child and adolescent development. Curr. Dir. Psychol. Sci 28 (3), 241–246. 10.1177/0963721419837670.

[R7] Belsky J, Steinberg L, Draper P, 1991. Childhood experience, interpersonal development, and reproductive strategy: an evolutionary theory of socialization. Child Dev. 62 (4), 647–670. 10.2307/1131166. 1935336

[R8] Bick J, Nelson CA, 2016. Early adverse experiences and the developing brain. Neuropsychopharmacology 41 (1), 177–196. 10.1038/npp.2015.252. 26334107 PMC4677140

[R9] Bloom PA, VanTieghem M, Gabard-Durnam L, Gee DG, Flannery J, Caldera C, Goff B, Telzer EH, Humphreys KL, Fareri DS, Shapiro M, Algharazi S, Bolger N, Aly M, Tottenham N, 2021. Age-related change in task-evoked amygdala-prefrontal circuitry: a multiverse approach with an accelerated longitudinal cohort aged 4–22 years, 10.08.463601. 10.1101/2021.10.08.463601, 2021. PMC9188973 35393752

[R10] Botvinik-Nezer R, Holzmeister F, Camerer CF, Dreber A, Huber J, Johannesson M, Kirchler M, Iwanir R, Mumford JA, Adcock RA, Avesani P, Baczkowski BM, Bajracharya A, Bakst L, Ball S, Barilari M, Bault N, Beaton D, Beitner J, , 2020. Variability in the analysis of a single neuroimaging dataset by many teams. Nature 582 (7810), 84–88. 10.1038/s41586-020-2314-9. 32483374 PMC7771346

[R11] Bowring A, Nichols TE, Maumet C, 2022. Isolating the sources of pipeline-variability in group-level task-fMRI results. Hum. Brain Mapp 43 (3), 1112–1128. 10.1002/hbm.25713. 34773436 PMC8764489

[R12] Bronfenbrenner U, Morris PA, 2007. The bioecological model of human development. In: Handbook of Child Psychology. American Cancer Society. 10.1002/9780470147658.chpsy0114.

[R13] Bryce NV, Flournoy JC, Guassi Moreira JF, Rosen ML, Sambook KA, Mair P, McLaughlin KA, 2021. Brain parcellation selection: an overlooked decision point with meaningful effects on individual differences in resting-state functional connectivity. Neuroimage 243, 118487. 10.1016/j.neuroimage.2021.118487. 34419594 PMC8629133

[R14] Callaghan BL, Tottenham N, 2016. The Stress Acceleration Hypothesis: effects of early-life adversity on emotion circuits and behavior. Current Opinion in Behavioral Sciences 7, 76–81. 10.1016/j.cobeha.2015.11.018. 29644262 PMC5890821

[R15] Casey BJ, Cannonier T, Conley MI, Cohen AO, Barch DM, Heitzeg MM, Soules ME, Teslovich T, Dellarco DV, Garavan H, Orr CA, Wager TD, Banich MT, Speer NK, Sutherland MT, Riedel MC, Dick AS, Bjork JM, Thomas KM, , 2018. The adolescent brain cognitive development (ABCD) study: imaging acquisition across 21 sites. Developmental Cognitive Neuroscience 32, 43–54. 10.1016/j.dcn.2018.03.001. 29567376 PMC5999559

[R16] Cheng TW, Magis-Weinberg L, Guazzelli Williamson V, Ladouceur CD, Whittle SL, Herting MM, Uban KA, Byrne ML, Barendse MEA, Shirtcliff EA, Pfeifer JH, 2021. A researcher’s guide to the measurement and modeling of puberty in the ABCD Study^®^ at baseline. Front. Endocrinol 12, 471. 10.3389/fendo.2021.608575. PMC8131843 34025573

[R17] Chilcoat HD, Anthony JC, 1996. Impact of parent monitoring on initiation of drug use through late childhood. J. Am. Acad. Child Adolesc. Psychiatry 35 (1), 91–100. 10.1097/00004583-199601000-00017. 8567618

[R18] Clawson A, Strang JF, Wallace GL, Gomez-Lobo V, Jack A, Webb SJ, Pelphrey KA, 2020. Parent-child concordance on the Pubertal Development Scale in typically developing and autistic youth. Research in Autism Spectrum Disorders 77, 101610. 10.1016/j.rasd.2020.101610. 32863862 PMC7449027

[R19] Cohen J, 1994. The earth is round (p < .05). Am. Psychol 49 (12), 997–1003. 10.1037/0003-066X.49.12.997.

[R20] Colich NL, Rosen ML, Williams ES, McLaughlin KA, 2020. Biological aging in childhood and adolescence following experiences of threat and deprivation: a systematic review and meta-analysis. Psychol. Bull 146 (9), 721–764. 10.1037/bul0000270. 32744840 PMC7484378

[R21] Conger RD, Conger KJ, Martin MJ, 2010. Socioeconomic status, family Processes, and individual development. J. Marriage Fam 72 (3), 685–704. 10.1111/j.1741-3737.2010.00725.x. 20676350 PMC2910915

[R22] Conger RD, Wallace LE, Sun Y, Simons RL, McLoyd VC, Brody GH, 2002. Economic pressure in African American families: a replication and extension of the family stress model. Dev. Psychol 38 (2), 179–193. 11881755

[R23] Corrás T, Seijo D, Fariña F, Novo M, Arce R, Cabanach RG, 2017. What and how much do children lose in academic settings owing to parental separation? Front. Psychol 8, 1545. 10.3389/fpsyg.2017.01545. 28955270 PMC5600987

[R24] De Bellis MD, Keshavan MS, Clark DB, Casey BJ, Giedd JN, Boring AM, Frustaci K, Ryan ND, 1999. Developmental traumatology part II: brain development**See accompanying Editorial, in this issue. Biol. Psychiatr 45 (10), 1271–1284. 10.1016/S0006-3223(99)00045-1. 10349033

[R25] DeJoseph ML, Herzberg MP, Sifre RD, Berry D, Thomas KM, 2022. Measurement matters: an individual differences examination of family socioeconomic factors, latent dimensions of children’s experiences, and resting state functional brain connectivity in the ABCD sample. Developmental Cognitive Neuroscience 53, 101043. 10.1016/j.dcn.2021.101043. 34915436 PMC8683693

[R26] Del Giacco AC, Jones SA, Morales AM, Kliamovich D, Nagel BJ, 2021. Adolescent novelty seeking is associated with greater ventral striatal and prefrontal brain response during evaluation of risk and reward. Cognit. Affect Behav. Neurosci 10.3758/s13415-021-00937-2. PMC8792307 34342865

[R27] Demidenko MI, 2022. Associated Code for an Open-Data Replication and Multiverse Analysis of an ABCD Study^®^: Mediating Effect of Pubertal Stages on the Family Environment and Neurodevelopment. 10.5281/zenodo.6819653. Zenodo. PMC9770593 36561641

[R28] Demidenko MI, Ip KI, Kelly DP, Constante K, Goetschius LG, Keating DP, 2021. Ecological stress, amygdala reactivity, and internalizing symptoms in preadolescence: is parenting a buffer? Cortex 140, 128–144. 10.1016/j.cortex.2021.02.032. 33984711 PMC8169639

[R29] Dick AS, Lopez DA, Watts AL, Heeringa S, Reuter C, Bartsch H, Fan CC, Kennedy DN, Palmer C, Marshall A, Haist F, Hawes S, Nichols TE, Barch DM, Jernigan TL, Garavan H, Grant S, Pariyadath V, Hoffman E, , 2021. Meaningful associations in the adolescent brain cognitive development study. Neuroimage 239, 118262. 10.1016/j.neuroimage.2021.118262. 34147629 PMC8803401

[R30] Dorn LD, Susman EJ, Ponirakis A, 2003. Pubertal timing and adolescent adjustment and behavior: conclusions vary by rater. J. Youth Adolesc 32 (3), 157–167. 10.1023/A:1022590818839.

[R31] Duffy KA, McLaughlin KA, Green PA, 2018. Early life adversity and health-risk behaviors: proposed psychological and neural mechanisms. Ann. N. Y. Acad. Sci 1428 (1), 151–169. 10.1111/nyas.13928. 30011075 PMC6158062

[R32] Ellis BJ, Garber J, 2000. Psychosocial antecedents of variation in girls’ pubertal timing: maternal depression, stepfather presence, and marital and family stress. Child Dev. 71 (2), 485–501. 10.1111/1467-8624.00159. 10834479

[R33] Ellis BJ, Shirtcliff EA, Boyce WT, Deardorff J, Essex MJ, 2011. Quality of early family relationships and the timing and tempo of puberty: effects depend on biological sensitivity to context. Dev. Psychopathol 23 (1), 85–99. 10.1017/S0954579410000660. 21262041 PMC3033698

[R34] Ellwood-Lowe ME, Whitfield-Gabrieli S, Bunge SA, 2021. Brain network coupling associated with cognitive performance varies as a function of a child’s environment in the ABCD study. Nat. Commun 12 (1), 7183. 10.1038/s41467-021-27336-y. 34893612 PMC8664837

[R35] Evans GW, Li D, Whipple SS, 2013. Cumulative risk and child development. Psychol. Bull 139 (6), 1342–1396. 10.1037/a0031808. 23566018

[R36] Farah MJ, 2017. The neuroscience of socioeconomic status: correlates, causes, and consequences. Neuron 96 (1), 56–71. 10.1016/j.neuron.2017.08.034. 28957676

[R37] Farah MJ, 2018. Socioeconomic status and the brain: prospects for neuroscience-informed policy. Nat. Rev. Neurosci 19 (7), 428–438. 10.1038/s41583-018-0023-2. 29867123

[R38] Flake JK, Luong R, Shaw M, 2022. Addressing a crisis of generalizability with large-scale construct validation. Behav. Brain Sci 45 10.1017/S0140525X21000376. 35139945

[R39] Fletcher SC, 2021. How (not) to measure replication. European Journal for Philosophy of Science 11 (2), 57. 10.1007/s13194-021-00377-2.

[R40] Flouri E, Midouhas E, Joshi H, 2014. Family poverty and trajectories of children’s emotional and behavioural problems: the moderating roles of self-regulation and verbal cognitive ability. J. Abnorm. Child Psychol 42 (6), 1043–1056. 10.1007/s10802-013-9848-3. 24473936

[R41] Forbes EE, Dahl RE, 2010. Pubertal development and behavior: hormonal activation of social and motivational tendencies. Brain Cognit. 72 (1), 66–72. 10.1016/j.bandc.2009.10.007. 19942334 PMC3955709

[R42] Funder DC, Ozer DJ, 2019. Evaluating effect size in psychological research: sense and nonsense. Advances in Methods and Practices in Psychological Science 2 (2), 156–168. 10.1177/2515245919847202.

[R43] Gach EJ, Ip KI, Sameroff AJ, Olson SL, 2018. Early cumulative risk predicts externalizing behavior at age 10: the mediating role of adverse parenting. J. Fam. Psychol.: JFP: Journal of the Division of Family Psychology of the American Psychological Association (Division 43) 32 (1), 92–102. 10.1037/fam0000360. 29543487

[R44] Garavan H, Bartsch H, Conway K, Decastro A, Goldstein RZ, Heeringa S, Jernigan T, Potter A, Thompson W, Zahs D, 2018. Recruiting the ABCD sample: design considerations and procedures. Developmental Cognitive Neuroscience 32, 16–22. 10.1016/j.dcn.2018.04.004. 29703560 PMC6314286

[R45] Gee DG, Humphreys KL, Flannery J, Goff B, Telzer EH, Shapiro M, Hare TA, Bookheimer SY, Tottenham N, 2013. A developmental shift from positive to negative connectivity in human amygdala–prefrontal circuitry. J. Neurosci 33 (10), 4584–4593. 10.1523/JNEUROSCI.3446-12.2013. 23467374 PMC3670947

[R46] Gelman A, Loken E, 2014. The statistical crisis in science: data-dependent analysis—a” garden of forking paths”—explains why many statistically significant comparisons don’t hold up. Am. Sci 102 (6), 460–466.

[R47] Gelman A, Stern H, 2006. The difference between “significant” and “not significant” is not itself statistically significant. Am. Statistician 60 (4), 328–331. 10.1198/000313006X152649.

[R48] Gonzalez MR, Palmer CE, Uban KA, Jernigan TL, Thompson WK, Sowell ER, 2020. Positive economic, psychosocial, and physiological ecologies predict brain structure and cognitive performance in 9–10-year-old children. Front. Hum. Neurosci 14, 436. 10.3389/fnhum.2020.578822. PMC7655980 33192411

[R49] Gonzalez R, Thompson EL, Sanchez M, Morris A, Gonzalez MR, Feldstein Ewing SW, Mason MJ, Arroyo J, Howlett K, Tapert SF, Zucker RA, 2021. An update on the assessment of culture and environment in the ABCD Study^®^: emerging literature and protocol updates over three measurement waves. Developmental Cognitive Neuroscience 52, 101021. 10.1016/j.dcn.2021.101021. 34700197 PMC8551602

[R50] Hackman DA, Farah MJ, Meaney MJ, 2010. Socioeconomic status and the brain: mechanistic insights from human and animal research. Nat. Rev. Neurosci 11 (9), 651–659. 10.1038/nrn2897. 20725096 PMC2950073

[R51] Hagler DJ, Hatton S, Cornejo MD, Makowski C, Fair DA, Dick AS, Sutherland MT, Casey BJ, Barch DM, Harms MP, Watts R, Bjork JM, Garavan HP, Hilmer L, Pung CJ, Sicat CS, Kuperman J, Bartsch H, Xue F, , 2019. Image processing and analysis methods for the adolescent brain cognitive development study. Neuroimage 202, 116091. 10.1016/j.neuroimage.2019.116091. 31415884 PMC6981278

[R52] Hair NL, Hanson JL, Wolfe BL, Pollak SD, 2015. Association of child poverty, brain development, and academic achievement. JAMA Pediatr. 169 (9), 822–829. 10.1001/jamapediatrics.2015.1475. 26192216 PMC4687959

[R53] Hanson JL, Hair N, Shen DG, Shi F, Gilmore JH, Wolfe BL, Pollak SD, 2013. Family poverty affects the rate of human infant brain growth. PLoS One 8 (12), e80954. 10.1371/journal.pone.0080954. 24349025 PMC3859472

[R54] Herting MM, Uban KA, Gonzalez MR, Baker FC, Kan EC, Thompson WK, Granger DA, Albaugh MD, Anokhin AP, Bagot KS, Banich MT, Barch DM, Baskin-Sommers A, Breslin FJ, Casey BJ, Chaarani B, Chang L, Clark DB, Cloak CC, , 2021. Correspondence between perceived pubertal development and hormone levels in 9–10 Year-olds from the adolescent brain cognitive development study. Front. Endocrinol 11, 1012. 10.3389/fendo.2020.549928. PMC7930488 33679599

[R55] Hodson G, 2021. Construct jangle or construct mangle? Thinking straight about (nonredundant) psychological constructs. Journal of Theoretical Social Psychology 5 (4), 576–590. 10.1002/jts5.120.

[R56] Hyde LW, Gard AM, Tomlinson RC, Burt SA, Mitchell C, Monk CS, 2020. An ecological approach to understanding the developing brain: examples linking poverty, parenting, neighborhoods, and the brain. Am. Psychol 75 (9), 1245–1259. 10.1037/amp0000741. 33382290 PMC8167378

[R57] Ip KI, Sisk LM, Horien C, Conley MI, Rapuano KM, Rosenberg MD, Greene AS, Scheinost D, Constable RT, Casey B, Baskin-Sommers A, Gee DG, 2022. Associations among household and neighborhood socioeconomic disadvantages, resting-state frontoamygdala connectivity, and internalizing symptoms in youth. J. Cognit. Neurosci 1–32. 10.1162/jocn_a_01826. 35104356

[R58] Irvine E, 2021. The role of replication studies in theory building. Perspect. Psychol. Sci 16 (4), 844–853. 10.1177/1745691620970558. 33440125

[R59] John LK, Loewenstein G, Prelec D, 2012. Measuring the prevalence of questionable research practices with incentives for truth telling. Psychol. Sci 23 (5), 524–532. 10.1177/0956797611430953. 22508865

[R60] Kapetanovic S, Boson K, 2020. Discrepancies in parents’ and adolescents’ reports on parent-adolescent communication and associations to adolescents’ psychological health. Curr. Psychol 10.1007/s12144-020-00911-0.

[R61] Kaufman J, Birmaher B, Brent D, Rao U, Flynn C, Moreci P, Williamson D, Ryan N, 1997. Schedule for affective Disorders and Schizophrenia for school-age children-present and lifetime version (K-SADS-PL): initial reliability and validity data. J. Am. Acad. Child Adolesc. Psychiatry 36 (7), 980–988. 10.1097/00004583-199707000-00021. 9204677

[R62] Kim K, Smith PK, 1998. Childhood stress, behavioural symptoms and mother–daughter pubertal development. J. Adolesc 21 (3), 231–240. 10.1006/jado.1998.0149. 9657891

[R63] Klapwijk ET, van den Bos W, Tamnes CK, Raschle NM, Mills KL, 2021. Opportunities for increased reproducibility and replicability of developmental neuroimaging. Developmental Cognitive Neuroscience 47, 100902. 10.1016/j.dcn.2020.100902. 33383554 PMC7779745

[R64] Lakens D, Adolfi FG, Albers CJ, Anvari F, Apps MAJ, Argamon SE, Baguley T, Becker RB, Benning SD, Bradford DE, Buchanan EM, Caldwell AR, Van Calster B, Carlsson R, Chen S-C, Chung B, Colling LJ, Collins GS, Crook Z, , 2018. Justify your alpha. Nat. Human Behav 2 (3), 168–171. 10.1038/s41562-018-0311-x.

[R65] Li X, Ai L, Giavasis S, Jin H, Feczko E, Xu T, Clucas J, Franco A, Heinsfeld AS, Adebimpe A, Vogelstein JT, Yan C-G, Esteban O, Poldrack RA, Craddock C, Fair D, Satterthwaite T, Kiar G, Milham MP, 2021. Moving beyond processing and analysis-related variation in neuroscience, 12.01.470790. 10.1101/2021.12.01.470790, 2021. 39103610

[R66] Linver MR, Brooks-Gunn J, Kohen DE, 2002. Family processes as pathways from income to young children’s development. Dev. Psychol 38 (5), 719–734. 10.1037/0012-1649.38.5.719. 12220050

[R67] Loken E, Gelman A, 2017. Measurement error and the replication crisis. Science 355 (6325), 584–585. 10.1126/science.aal3618. 28183939

[R68] Ly A, Etz A, Marsman M, Wagenmakers E-J, 2019. Replication Bayes factors from evidence updating. Behav. Res. Methods 51 (6), 2498–2508. 10.3758/s13428-018-1092-x. 30105445 PMC6877488

[R69] Mackinnon DP, Dwyer JH, 1993. Estimating mediated effects in prevention studies. Eval. Rev 17 (2), 144–158. 10.1177/0193841X9301700202.

[R70] Marek S, Tervo-Clemmens B, Calabro FJ, Montez DF, Kay BP, Hatoum AS, Donohue MR, Foran W, Miller RL, Hendrickson TJ, Malone SM, Kandala S, Feczko E, Miranda-Dominguez O, Graham AM, Earl EA, Perrone AJ, Cordova M, Doyle O, , 2022. Reproducible brain-wide association studies require thousands of individuals. Nature 1–7. 10.1038/s41586-022-04492-9. PMC8991999 35296861

[R71] McEwen BS, Akil H, 2020. Revisiting the stress concept: implications for affective Disorders. J. Neurosci.: J. Soc. Neurosci 40 (1), 12–21. 10.1523/JNEUROSCI.0733-19.2019. PMC6939488 31896560

[R72] McLaughlin KA, Sheridan MA, Humphreys KL, Belsky J, Ellis BJ, 2021. The value of dimensional models of early experience: thinking clearly about concepts and categories. Perspect. Psychol. Sci 16 (6), 1463–1472. 10.1177/1745691621992346. 34491864 PMC8563369

[R73] McLaughlin KA, Sheridan MA, Lambert HK, 2014. Childhood adversity and neural development: deprivation and threat as distinct dimensions of early experience. Neurosci. Biobehav. Rev 47, 578–591. 10.1016/j.neubiorev.2014.10.012. 25454359 PMC4308474

[R74] McLaughlin KA, Sheridan MA, Nelson CA, 2017. Neglect as a violation of species-expectant experience: neurodevelopmental consequences. Biol. Psychiatr 82 (7), 462–471. 10.1016/j.biopsych.2017.02.1096. PMC5572554 28392082

[R75] McLoyd VC, 1998. Socioeconomic disadvantage and child development. Am. Psychol 53 (2), 185–204. 10.1037//0003-066x.53.2.185. 9491747

[R76] McNeilly EA, Saragosa-Harris N, Mills K, Dahl R, Magis-Weinberg L, 2021. Reward sensitivity and internalizing symptoms during the transition to puberty: an examination of 9-and 10-year-olds in the ABCD Study. PsyArXiv. 10.31234/osf.io/6ebuq. PMC9649995 36368089

[R77] McNeish D, Wolf MG, 2021. Dynamic fit index cutoffs for confirmatory factor analysis models. Psychological Methods, No Pagination Specified-No Pagination Specified. 10.1037/met0000425. 34694832

[R78] Mersky JP, Janczewski CE, Topitzes J, 2017. Rethinking the measurement of adversity: moving toward second-generation research on adverse childhood experiences. Child. Maltreat 22 (1), 58–68. 10.1177/1077559516679513. 27920222

[R79] Mischel W, 2008. The toothbrush problem. APS Observer 21 (11). https://www.psychologicalscience.org/observer/the-toothbrush-problem.

[R80] Moffitt TE, Caspi A, Belsky J, Silva PA, 1992. Childhood experience and the onset of menarche: a test of a sociobiological model. Child Dev. 63 (1), 47–58. 10.2307/1130900. 1551329

[R81] Moos RH, Moos BS, 1976. A typology of family social environments. Fam. Process 15 (4), 357–371. 10.1111/j.1545-5300.1976.00357.x. 1026454

[R82] Open Science Collaboration, 2015. Estimating the reproducibility of psychological science. Science 349 (6251), aac4716. 10.1126/science.aac4716. 26315443

[R83] Orben A, Dienlin T, Przybylski AK, 2019. Social media’s enduring effect on adolescent life satisfaction. Proc. Natl. Acad. Sci. USA 116 (21), 10226–10228. 10.1073/pnas.1902058116. 31061122 PMC6534991

[R84] Oshri A, Duprey EK, Liu S, Gonzalez A, 2020. Chapter 14 - ACEs and resilience: methodological and conceptual issues. In: Asmundson GJG, Afifi TO (Eds.), Adverse Childhood Experiences. Academic Press, pp. 287–306. 10.1016/B978-0-12-816065-7.00014-8.

[R85] Owens MM, Potter A, Hyatt CS, Albaugh M, Thompson WK, Jernigan T, Yuan D, Hahn S, Allgaier N, Garavan H, 2021. Recalibrating expectations about effect size: a multi-method survey of effect sizes in the ABCD study. PLoS One 16 (9), e0257535. 10.1371/journal.pone.0257535. 34555056 PMC8460025

[R86] Park AT, Leonard JA, Saxler PK, Cyr AB, Gabrieli JDE, Mackey AP, 2018. Amygdala–medial prefrontal cortex connectivity relates to stress and mental health in early childhood. Soc. Cognit. Affect Neurosci 13 (4), 430–439. 10.1093/scan/nsy017. 29522160 PMC5928403

[R87] Petersen AC, Crockett L, Richards M, Boxer A, 1988. A self-report measure of pubertal status: reliability, validity, and initial norms. J. Youth Adolesc 17 (2), 117–133. 10.1007/BF01537962. 24277579

[R88] Petrican R, Miles S, Rudd L, Wasiewska W, Graham KS, Lawrence AD, 2021. Pubertal timing and functional neurodevelopmental alterations independently mediate the effect of family conflict on adolescent psychopathology. Developmental Cognitive Neuroscience 52, 101032. 10.1016/j.dcn.2021.101032. 34781251 PMC10436252

[R89] Pizzagalli DA, 2014. Depression, stress, and anhedonia: toward a synthesis and integrated model. Annu. Rev. Clin. Psychol 10, 393–423. 10.1146/annurev-clinpsy-050212-185606. 24471371 PMC3972338

[R90] Pollak SD, Smith KE, 2021. Thinking clearly about biology and childhood adversity: next steps for continued progress. Perspect. Psychol. Sci 16 (6), 1473–1477. 10.1177/17456916211031539. 34491865 PMC8564234

[R91] Proulx T, Morey RD, 2021. Beyond statistical ritual: theory in psychological science. Perspect. Psychol. Sci.: A Journal of the Association for Psychological Science 16 (4), 671–681. 10.1177/17456916211017098. 34240651

[R92] R Core Team, 2020. R: A Language and Environment for Statistical Computing. R Foundation for Statistical Computing. https://www.R-project.org/.

[R93] Rakesh D, Seguin C, Zalesky A, Cropley V, Whittle S, 2021a. Associations between neighborhood disadvantage, resting-state functional connectivity, and behavior in the adolescent brain cognitive development study: the moderating role of positive family and school environments. Biol. Psychiatr.: Cognitive Neuroscience and Neuroimaging 6 (9), 877–886. 10.1016/j.bpsc.2021.03.008. 33771727

[R94] Rakesh D, Zalesky A, Whittle S, 2021b. Similar but distinct – effects of different socioeconomic indicators on resting state functional connectivity: findings from the Adolescent Brain Cognitive Development (ABCD) Study. Developmental Cognitive Neuroscience 51, 101005. 10.1016/j.dcn.2021.101005. 34419766 PMC8379618

[R95] Repetti RL, Taylor SE, Seeman TE, 2002. Risky families: family social environments and the mental and physical health of offspring. Psychol. Bull 128 (2), 330–366. 11931522

[R96] Rijnhart JJM, Twisk JWR, Deeg DJH, Heymans MW, 2021. Assessing the Robustness of Mediation Analysis Results Using Multiverse Analysis. Prevention Science. 10.1007/s11121-021-01280-1. PMC9283158 34272641

[R97] Rohrer JM, Hünermund P, Arslan RC, Elson M, 2021. That’s a lot to process! Pitfalls of popular path models. PsyArXiv. 10.31234/osf.io/paeb7.

[R98] Romero F, 2019. Philosophy of science and the replicability crisis. Philos. Compass 14 (11), e12633. 10.1111/phc3.12633.

[R99] Rosseel Y, 2012. Lavaan: an R package for structural equation modeling. J. Stat. Software 48 (1), 1–36. 10.18637/jss.v048.i02.

[R100] Rosseel Y, Jorgensen TD, Rockwood N, Oberski D, Byrnes J, Vanbrabant L, Savalei V, Merkle E, Hallquist M, Rhemtulla M, Katsikatsou M, Barendse M, Scharf F, Du H, 2021. Lavaan: Latent Variable Analysis [Computer software], 0.6–9. https://CRAN.R-project.org/package=lavaan.

[R101] Rubin M, 2021. When to adjust alpha during multiple testing: a consideration of disjunction, conjunction, and individual testing. Synthese. 10.1007/s11229-021-03276-4.

[R102] Schaefer ES, 1965. Children’s reports of parental behavior: an inventory. Child Dev. 36 (2), 413–424. 10.2307/1126465. 14300862

[R103] Simmons JP, Nelson LD, Simonsohn U, 2011. False-positive psychology: undisclosed flexibility in data collection and analysis allows presenting anything as significant. Psychol. Sci 22 (11), 1359–1366. 10.1177/0956797611417632. 22006061

[R104] Simonsohn U, Nelson LD, Simmons JP, 2014. P-curve: a key to the file-drawer. J. Exp. Psychol. Gen 143 (2), 534–547. 10.1037/a0033242. 23855496

[R105] Simonsohn U, Simmons JP, Nelson LD, 2020. Specification curve analysis. Nat. Human Behav 1 10.1038/s41562-020-0912-z. -7. 32719546

[R106] Smith KE, Pollak SD, 2020. Rethinking concepts and categories for understanding the neurodevelopmental effects of childhood adversity. Perspect. Psychol. Sci 1745691620920725 10.1177/1745691620920725. PMC7809338 32668190

[R107] Sripada C, Angstadt M, Taxali A, Clark DA, Greathouse T, Rutherford S, Dickens JR, Shedden K, Gard AM, Hyde LW, Weigard A, Heitzeg M, 2021. Brain-wide functional connectivity patterns support general cognitive ability and mediate effects of socioeconomic status in youth. Transl. Psychiatry 11 (1), 1–8. 10.1038/s41398-021-01704-0. 34750359 PMC8575890

[R108] Steegen S, Tuerlinckx F, Gelman A, Vanpaemel W, 2016. Increasing transparency through a multiverse analysis: perspectives on psychological science. 10.1177/1745691616658637. 27694465

[R109] Taylor RL, Cooper SR, Jackson JJ, Barch DM, 2020. Assessment of neighborhood poverty, cognitive function, and prefrontal and hippocampal volumes in children. JAMA Netw. Open 3 (11). 10.1001/jamanetworkopen.2020.23774. PMC7610187 33141160

[R110] Thijssen S, Collins PF, Luciana M, 2020. Pubertal development mediates the association between family environment and brain structure and function in childhood. Dev. Psychopathol 32 (2), 687–702. 10.1017/S0954579419000580. 31258099 PMC7525116

[R111] Thijssen S, Collins PF, Luciana M, 2021. Pubertal development mediates the association between family environment and brain structure and function in childhood – addendum. Dev. Psychopathol 33 (1), 372–375. 10.1017/S0954579420000322. PMC7525116 31258099

[R112] Thijssen S, Collins PF, Luciana M, 2022. Does pubertal stage mediate the association between family environment and structure and function of the amygdala-mPFC circuit? A replication study of the longitudinal ABCD cohort. Developmental Cognitive Neuroscience 56, 101120. 10.1016/j.dcn.2022.101120. 35716638 PMC9213703

[R113] Thijssen S, Muetzel RL, Bakermans-Kranenburg MJ, Jaddoe VWV, Tiemeier H, Verhulst FC, White T, Ijzendoorn MHV, 2017. Insensitive parenting may accelerate the development of the amygdala–medial prefrontal cortex circuit. Dev. Psychopathol 29 (2), 505–518. 10.1017/S0954579417000141. 28401836

[R114] Thompson WH, Wright J, Bissett PG, 2020. Open exploration. Elife 9, e52157. 10.7554/eLife.52157. 31916934 PMC6964967

[R115] Tooley UA, Bassett DS, Mackey AP, 2021. Environmental influences on the pace of brain development. Nat. Rev. Neurosci 22 (6), 372–384. 10.1038/s41583-021-00457-5. 33911229 PMC8081006

[R116] Volkow ND, Koob GF, Croyle RT, Bianchi DW, Gordon JA, Koroshetz WJ, Pérez-Stable EJ, Riley WT, Bloch MH, Conway K, Deeds BG, Dowling GJ, Grant S, Howlett KD, Matochik JA, Morgan GD, Murray MM, Noronha A, Spong CY, , 2018. The conception of the ABCD study: from substance use to a broad NIH collaboration. Developmental Cognitive Neuroscience 32, 4–7. 10.1016/j.dcn.2017.10.002. 29051027 PMC5893417

[R117] Wagenmakers E-J, 2007. A practical solution to the pervasive problems ofp values. Psychon. Bull. Rev 14 (5), 779–804. 10.3758/BF03194105. 18087943

[R118] Whittle S, Simmons JG, Dennison M, Vijayakumar N, Schwartz O, Yap MBH, Sheeber L, Allen NB, 2014. Positive parenting predicts the development of adolescent brain structure: a longitudinal study. Developmental Cognitive Neuroscience 8, 7–17. 10.1016/j.dcn.2013.10.006. 24269113 PMC6990097

[R119] Yi-Frazier JP, Hilliard ME, Fino NF, Naughton MJ, Liese AD, Hockett CW, Hood KK, Pihoker C, Seid M, Lang W, Lawrence JM, 2016. Whose quality of life is it anyway? Discrepancies between youth and parent health-related quality of life ratings in type 1 and type 2 diabetes. Qual. Life Res.: An International Journal of Quality of Life Aspects of Treatment, Care and Rehabilitation 25 (5), 1113–1121. 10.1007/s11136-015-1158-5. PMC4936832 26466834

[R120] Zucker RA, Gonzalez R, Feldstein Ewing SW, Paulus MP, Arroyo J, Fuligni A, Morris AS, Sanchez M, Wills T, 2018. Assessment of culture and environment in the adolescent brain and cognitive development study: rationale, description of measures, and early data. Developmental Cognitive Neuroscience 32, 107–120. 10.1016/j.dcn.2018.03.004. 29627333 PMC6436615

[R121] Zuo P, Wang Y, Liu J, Hu S, Zhao G, Huang L, Lin D, 2019. Effects of early adversity on the brain: larger-volume anterior cingulate cortex in AIDS orphans. PLoS One 14 (1), e0210489. 10.1371/journal.pone.0210489. 30640928 PMC6331092

[R122] Zwaan RA, Etz A, Lucas RE, Donnellan MB, 2018. Making Replication Mainstream, vol. 41. Behavioral and Brain Sciences. 10.1017/S0140525X17001972. 29065933

