# Supplementary material for: Mediating effect of pubertal stages on the family environment and neurodevelopment: An open-data replication and multiverse analysis of an ABCD Study®
Source: Neuroimage Rep. 2022 Sep 18;2(4):100133. doi: 10.1016/j.ynirp.2022.100133 (PMC9770593; doi:10.1016/j.ynirp.2022.100133)
Supplement: Multimedia component 1 [file mmc1.docx]

## Measures

## Environmental Measures

### *Child Items/Factor*

An abbreviated measure of maternal acceptance of the *Child Report of Parent Behavior Inventory* (CRPBI; Schaefer, 1965) is used. The 5-item CRPBI is used to assess children’s perception of the parent-child dyadic relationship, with a strong emphasis on emotional support from parents. It includes statements such as “Makes me feel better after talking about my worries with him/her” and “Is able to make me feel better when I am upset” which were rated on a 3-point Likert scale ranging from (1) “Not like him/her” to (3) “A lot like him/her”. For the individual item scale, items are averaged such that higher scores indicate higher perceptions of parental acceptance.

The youth reported *Family Environment Scale* (FES-Y; Moos & Moos, 1976) is a youth self-report measure that assesses family social environment as perceived by the family member. The FES-Y is a 9-item questionnaire that ascribes the level of conflict within the family using statements such as “We fight a lot in our family” or “Family members often criticize each other” which the participant either responds to as true or false. For the individual item scale, items are averaged and reverse coded such that higher scores indicate lower perceptions of family conflict.

The *Parental Monitoring Survey* (PMON; Chilcoat & Anthony, 1996) is a youth self-report measure that assesses parental monitoring/supervision. The 5-item PMON measures how much the youth believes the parent monitors their whereabouts and includes statements such as “How often do your parents know who you are with when you are not at school and away from home” or “How often do your parents/guardians know where you are?”, which were rated on a 5-point Likert scale ranging from (1) “Never” to (5) “Always or Almost Always”. For the individual item scale, items are averaged such that higher scores indicate higher parental monitoring.

### *Parent Items/Factor*

Similar to the FES-Y, the parent-reported *Family Environment Scale* (FES-P) is a measure that assesses family social environment as perceived by the family member. The FES-P is a 9-item questionnaire that ascribes the level of conflict within the family using statements such as “We fight a lot in our family” or “Family members often criticize each other”, which the participant either responds to as true or false. For the individual item scale, items are averaged and reverse coded such that higher scores indicate lower perceptions of family conflict.

One item measuring conflict from the *Kiddie Schedule for Affective Disorders and Schizophrenia* (KSADS; Kaufman et al., 1997) was used to assess parental-child conflict. This one item measured the dynamic relationship with the question “In general, how do you and your child get along?”, using a 3-point Likert scale ranging from (1) “Very Well” to (3) “A lot of conflict”. High scores on this item indicate a more negative relationship between the child and parent.

### *Demographic Items/Factor*

Youths self-reported their age in months, their sex at birth using options “Male”, “Female”, “Other”, and race/ethnicity, (1) “White, (2) “Black”, (3) “Hispanic”, (4) “Asian”, (5) “Other”. These variables were used as covariates in the mediation models, consistent with Thijssen et al. (2020).

Parents self-reported on income, education, separation and pregnancy variables. For combined household income, parents selected an income category for the past 12 months ranging from (1) “less than $5,000” to (10) “$200,000 and greater”, with the option to select “refuse to answer” or “don’t know”. Parents reported on their or their partner's highest level of education by selecting an education category that ranged from (0) “Never Attended” to (21) “Doctoral Degree”, with the option to “refuse to answer” and “don’t know”. Parents reported on their marital status, such as (1) “Married” or (6) “Living with partner” and whether their pregnancy with the child was a planned pregnancy (Yes/No).

Parental psychopathology was assessed using the comprehensive measure from the Achenbach System of Empirically Based Assessment Adult Self-Report (ASRS; Achenbach & Rescorla, 2003). The ASRS assesses different levels of psychopathology, such as anxiety, depression, withdrawal, thought problems and somatic complaints. Here, the t-scored (related to gender at ages 18-35 and 36-59 based on national probability samples) total problems score is used (range 25 to 100), whereby higher values relate to higher problems.

## Pubertal Stage

The *Pubertal Development Scale* (PDS; Petersen et al., 1988) assesses the child’s pubertal stage. The PDS is a non-invasive measure that assesses current pubertal status in females and males. For females, items assess changes in 1) body hair, 2) breast development and 3) menstruation. For males, items assess changes in 1) body hair, 2) hair on the face and 3) deepening voice. In the ABCD study, there are both youth and parent reported PDS scores. For the individual item scale, items are averaged such that the pubertal category score provides a 1-5 reference for whether a participant is: 1 = “pre puberty”; 2 = “early puberty”; 3 = “mid puberty”; 4 = “late puberty”; or 5 = “post puberty”., whereby higher scores indicate further progression in puberty.

## Analysis

The mediation model is composed of several parts: path-c, path-a, path-b, path-c’ and the indirect effect, as illustrated in traditional mediation analyses (Baron & Kenny, 1986; Mackinnon & Dwyer, 1993) and shown in-text Figure 1. Path-c is the total effect of the independent variable (IV) exerted onto the dependent variable (DV); path-a is the effect of the IV on the mediator; path-b is the effect of the mediator on the DV adjusted for the IV; and path-c’, or the direct effect, is the effect of the IV on the DV adjusted for the effects of path-a and path-b, respectively. The effect of the mediator, or the effect of the IV on DV through the mediator M, is the product of path-a and path-b. For each given model, the total effect (c) is equal to the combination of the direct effect (c’) and indirect effect (ab). Given the skew and kurtosis of the indirect effect, R implements bootstrapping to obtain bias-corrected confidence intervals and *p*-values (Hayes, 2009). For each model, bootstrapping is held constant at 5,000 sample permutations.

## Results

### *Distributions, Descriptives, Correlations for Variables*

*Table S1:*

Pearson’s Correlations Among Self-report and Brain Variables

|  | **1** | **2** | **3** | **4** | **5** | **6** | **7** | **8** | **9** | **10** | **11** | **12** | **13** | **14** | **15** | **16** | **17** | **18** | **19** |
| --- | --- | --- | --- | --- | --- | --- | --- | --- | --- | --- | --- | --- | --- | --- | --- | --- | --- | --- | --- |
| 1. Age | - |  |  |  |  |  |  |  |  |  |  |  |  |  |  |  |  |  |  |
| 2. Sex | .03 | - |  |  |  |  |  |  |  |  |  |  |  |  |  |  |  |  |  |
| 3. FamEnv Fact | .04 | -.08 | - | **.67** | **.76** | **.80** |  |  |  |  |  |  |  |  |  |  |  |  |  |
| 4. Demo Fact | .01 | .01 | .64 | - | **.31** | **.29** |  |  |  |  |  |  |  |  |  |  |  |  |  |
| 5. Child Fact | .07 | -.15 | .59 | .13 | - | **.38** |  |  |  |  |  |  |  |  |  |  |  |  |  |
| 6. Par Fact | -.01 | -.04 | .70 | .13 | .13 | - |  |  |  |  |  |  |  |  |  |  |  |  |  |
| 7. FES Yth | .06 | -.06 | .54 | .15 | .57 | .22 | - |  |  |  |  |  |  |  |  |  |  |  |  |
| 8. FES Par | -.01 | -.03 | .64 | .09 | .12 | .94 | -.20 | - |  |  |  |  |  |  |  |  |  |  |  |
| 9. PMON | .10 | -.17 | .41 | .15 | .70 | .04 | .24 | .05 | 1.00 |  |  |  |  |  |  |  |  |  |  |
| 10. Par Accept | .01 | -.05 | .40 | .06 | .81 | .08 | .29 | .07 | .36 | - |  |  |  |  |  |  |  |  |  |
| 11. Avg IncEdu | .01 | .01 | .59 | .95 | .12 | .11 | .14 | .07 | .14 | .05 | - |  |  |  |  |  |  |  |  |
| 12. PDS Par | .18 | -.48 | -.13 | -.24 | .04 | -.04 | .00 | -.03 | .06 | -.02 | -.19 | - |  |  |  |  |  |  |  |
| 13. PDS Yth | .12 | -.19 | -.13 | -.17 | -.07 | -.02 | -.08 | .00 | -.03 | -.05 | -.15 | .43 | - |  |  |  |  |  |  |
| 14. PDS ParYth | .18 | -.42 | -.14 | -.23 | -.01 | -.03 | -.04 | -.02 | .03 | -.04 | -.19 | .85 | .84 | - |  |  |  |  |  |
| 15. Amyg Vol | .08 | .14 | -.02 | .00 | -.01 | -.02 | .00 | -.02 | -.02 | .00 | .00 | -.04 | -.01 | -.03 | - | **.07** | **-.02** | **-.05** | **-.03** |
| 16. ACC CT | -.17 | -.06 | -.01 | .01 | -.03 | .00 | -.01 | .00 | -.04 | -.01 | .03 | -.04 | -.01 | -.02 | -.04 | - | **-.08** | **-.01** | **.03** |
| 17. ACC CA | .14 | .04 | -.01 | -.04 | .04 | -.01 | .00 | .00 | .03 | .04 | -.04 | .04 | .01 | .02 | .08 | -.44 | - | **.02** | **.00** |
| 18. L AmygCON | .00 | .03 | .11 | .16 | .04 | .03 | .04 | .02 | .03 | .03 | .14 | -.09 | -.04 | -.08 | .01 | -.01 | -.02 | - | **.58** |
| 19. R AmygCON | .00 | -.01 | .09 | .12 | .04 | .01 | .05 | .01 | .02 | .03 | .12 | -.06 | -.03 | -.06 | -.02 | .02 | -.03 | .57 | - |

​​Note: Lower diagonal values are Pearson *r* values from the current study. The upper diagonal **bold yellow** values are correlation values reported in Tables 3 & 4 (pg 694) in Thijssen et al. (2020).

Abbreviations: FamEnv = Family Environment; Demo = Demographic; Fact = Factor; Par = Parent; Yth = Youth; FES = Family Environment Scale (i.e. Conflict; reverse coded); PMON = Parental Monitoring; Accept = Child Report of Parent Behavior Inventory (i.e., Acceptance); Avg IncEdu = Average Parent Reported Income & Education; PDS = Pubertal Development Scale; Amyg = Amygdala; ACC = Anterior Cingulate Cortex; CT = Cortical Thickness; CA = Cortical Area; L/R AmygCON = Left/Right Amygdala Cingulo-Opercular Network connectivity


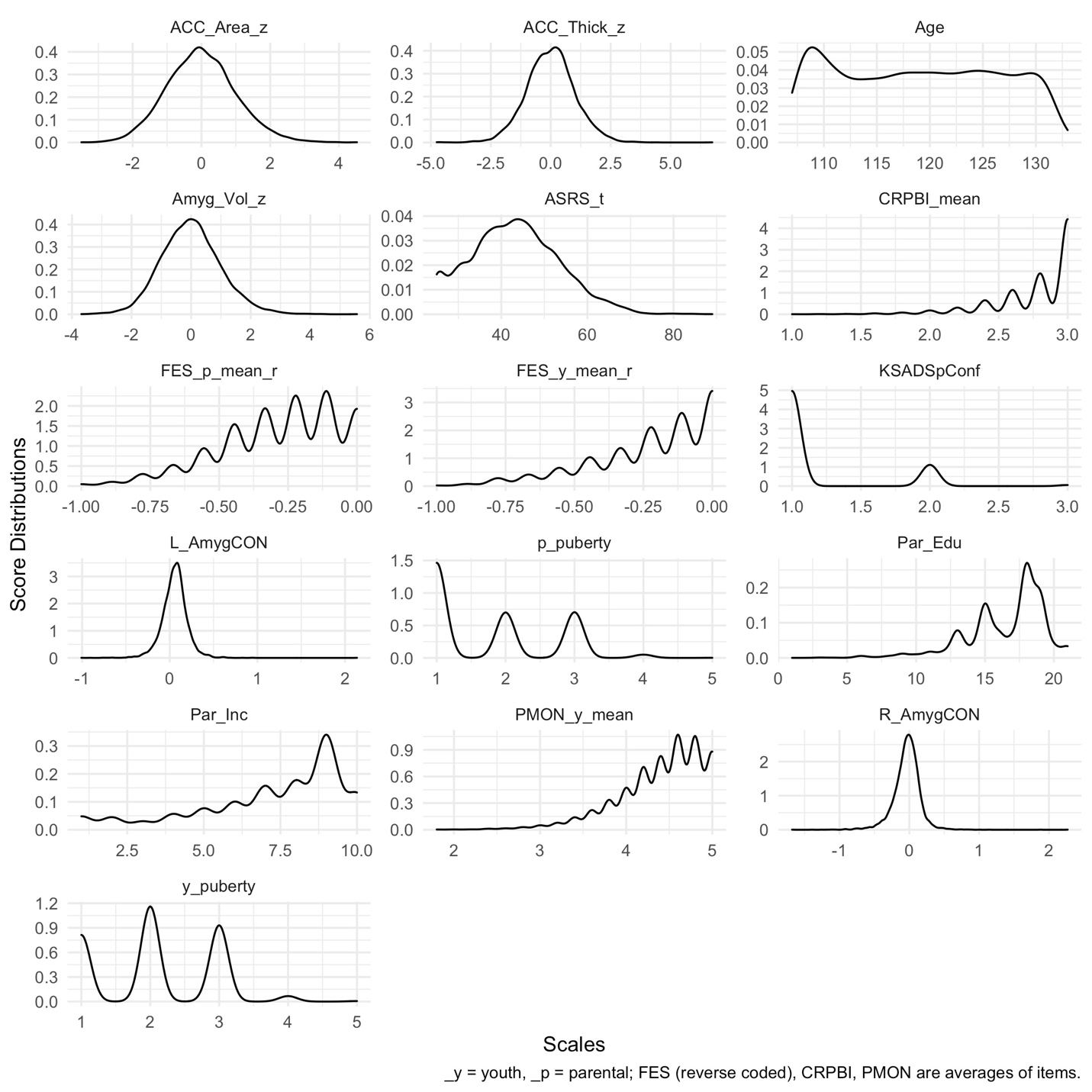


*Figure S1.* Distributions for several key variables

##

### *Aim 1: Replication Results*

Here, we extend the subjective ratings of replication for mediation of the direct and indirect paths from the manuscript, with the a-path, b-path, and total path. Same raters reported whether the effect (1) did or did not (0) replicate. Across the ratings, 70% agreed that the effects replicated. Specifically, all the effects in a-path were considered to replicate (100% agreement), except for less consensus with Amygdala volume (60% agreement), due likely to what may be a discrepancy in the effect reported in the original studies table/figure–we used the table that had a minor typo. For b-path, raters agreed that the effects replicated for Amygdala volume and ACC CT (100% agreement), less consensus for Left/Right AmygCON (60% agreement) and ACC CA did not replicate (100% agreement). For total effects, raters agreed that the Left AmygCON replicated (100% agreement), less consensus about the Right Amygdala volume (60% agreed it did not replicate) and most agreed that effects of ACC CA and ACC CT did not replicate (80% agreement).
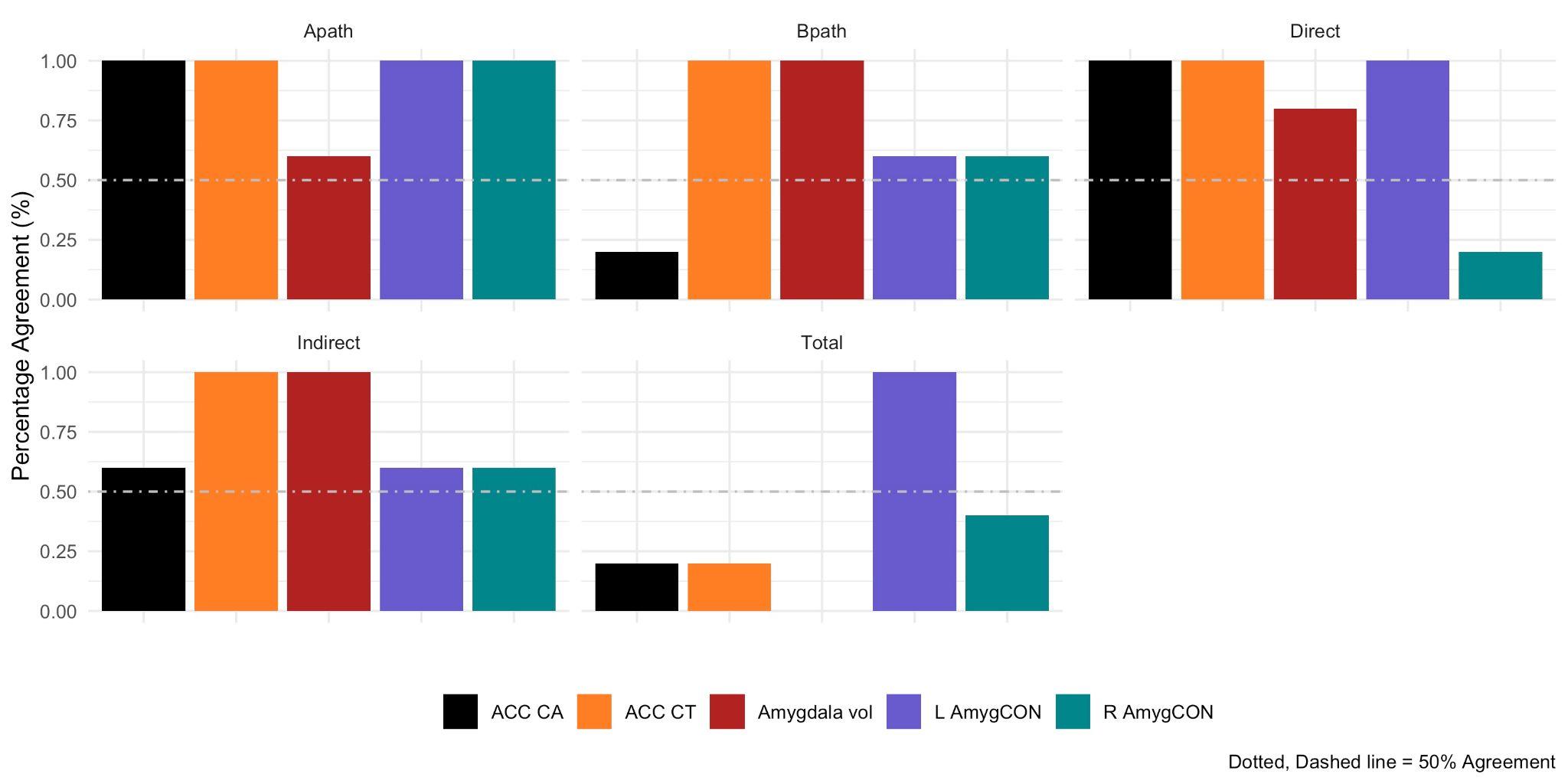
*Figure S2*. Reported Agreement of Replication of Effects Between Thijssen et al (2020) and this Replication Study.

ACC = Anterior Cingulate Cortex; CA = Cortical Area; CT = Cortical Thickness; L/R AmygCON = Left/Right Amygdala Cingulo-Opercular Network connectivity

The figure below displays the overlap between the beta estimate and its 95% confidence interval **from Thijssen et al. (2020**) relative to the replicated effect in this study


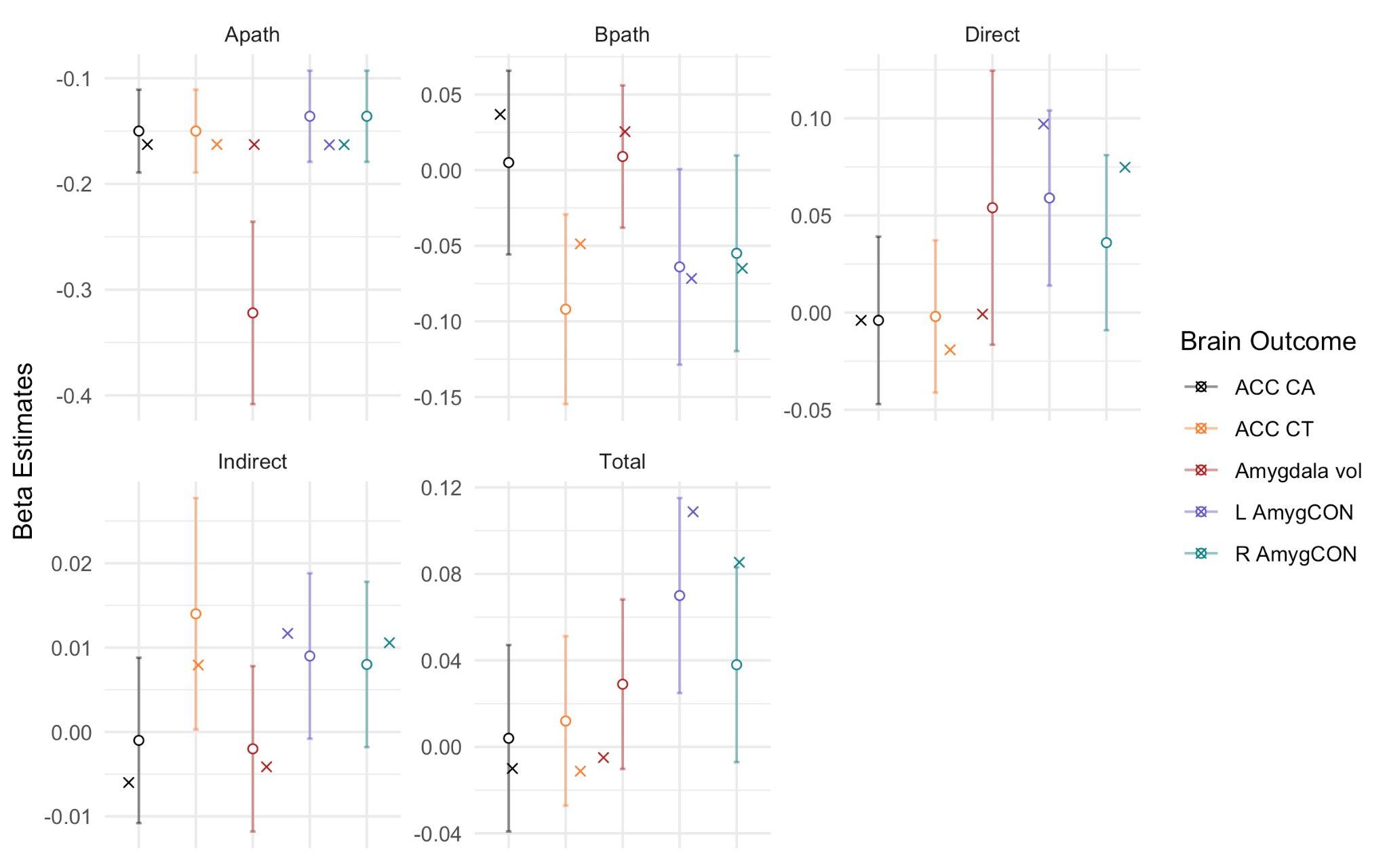


*Figure S3.* Reported standardized β estimates for A-path, B-path, Direct, Indirect and Total effects from original study by Thijssen et al. (2020) and replication study.

Original Study with associated 95% CI = ○; Replication Study = ✕.

ACC = Anterior Cingulate Cortex; CA = Cortical Area; CT = Cortical Thickness; Vol = Volume; L/R AmygCON = Left/Right Amygdala Cingulo-Opercular Network connectivity

Opposite of the above, the figure below displays the overlap between the beta estimate and its 95% confidence interval from **the replication study** relative to the original effect in Thijssen et al. (2020).


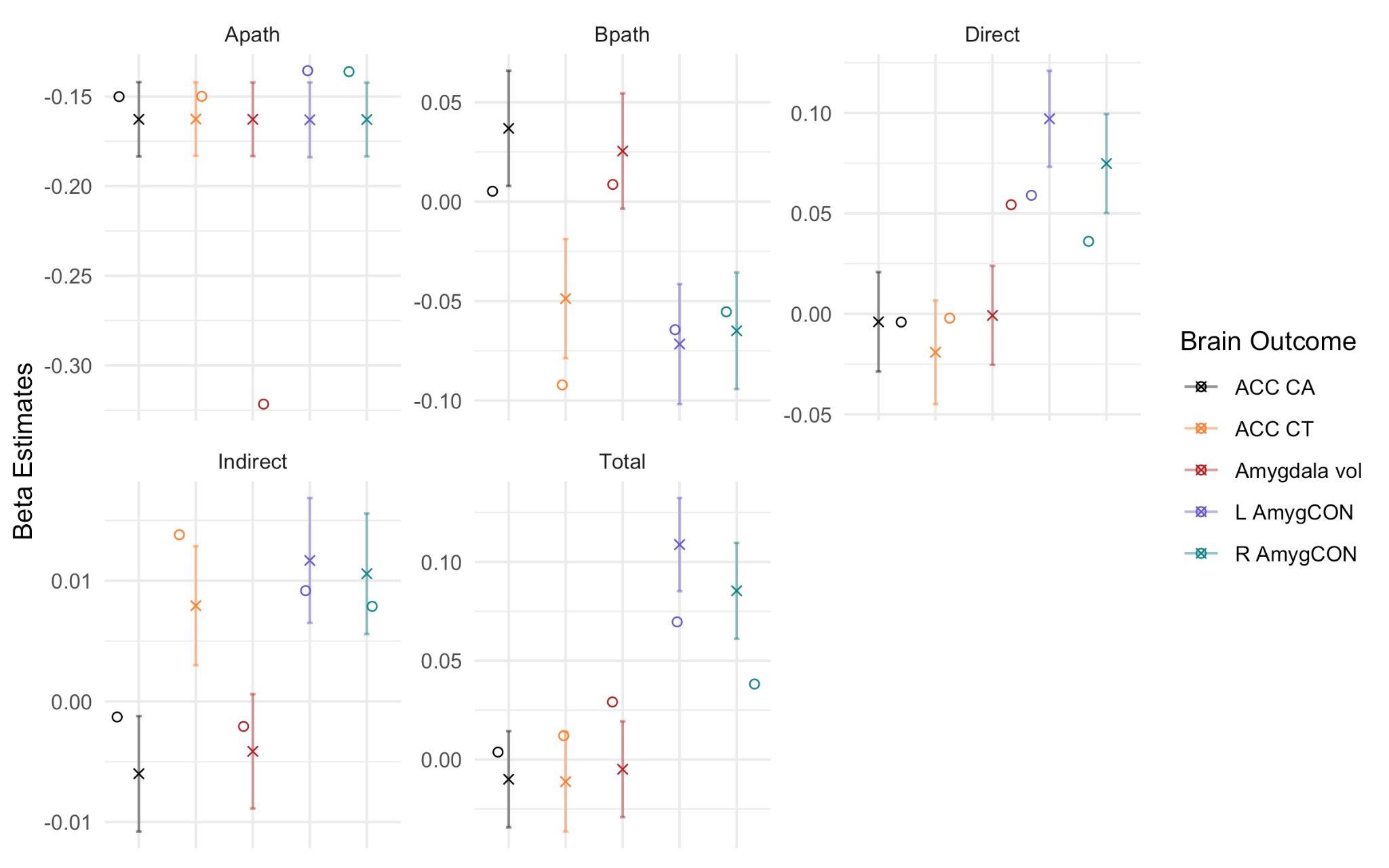


*Figure S4.* Reported standardized β estimates for A-path, B-path, Direct, Indirect and Total effects from original study by Thijssen et al. (2020) and Replication study.

Original Study = ○; Replication Study with associated 95% CI = ✕.

ACC = Anterior Cingulate Cortex; CA = Cortical Area; CT = Cortical Thickness; Vol = Volume; L/R AmygCON = Left/Right Amygdala Cingulo-Opercular Network connectivity

The bar plot below summarizes whether the beta estimates between the replication and the original are in the same direction and have the same category p-value. In addition, it compares the overlap between estimates and the 95% confidence intervals described above. The figure below represents the direct and indirect effect paths.
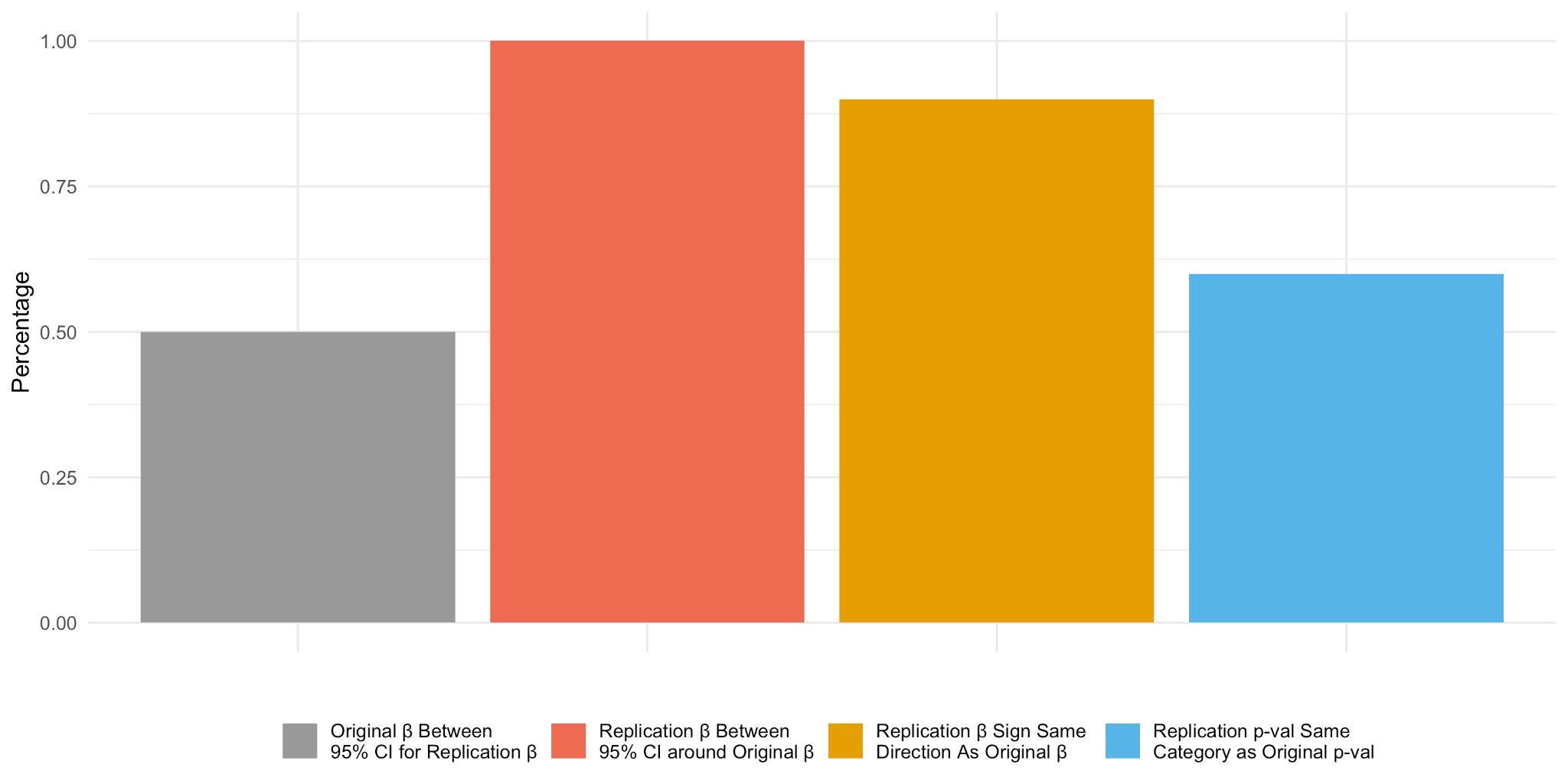


*Figure S5.* Summary of overlap in significance, 95% Confidence Interval, and β estimate direction between original study (Thijseen et al., 2020) and replication study for Direct and Indirect Effects.

##

### *Aim 2: Multiverse Results*

Multiverse, **Total Effects:**


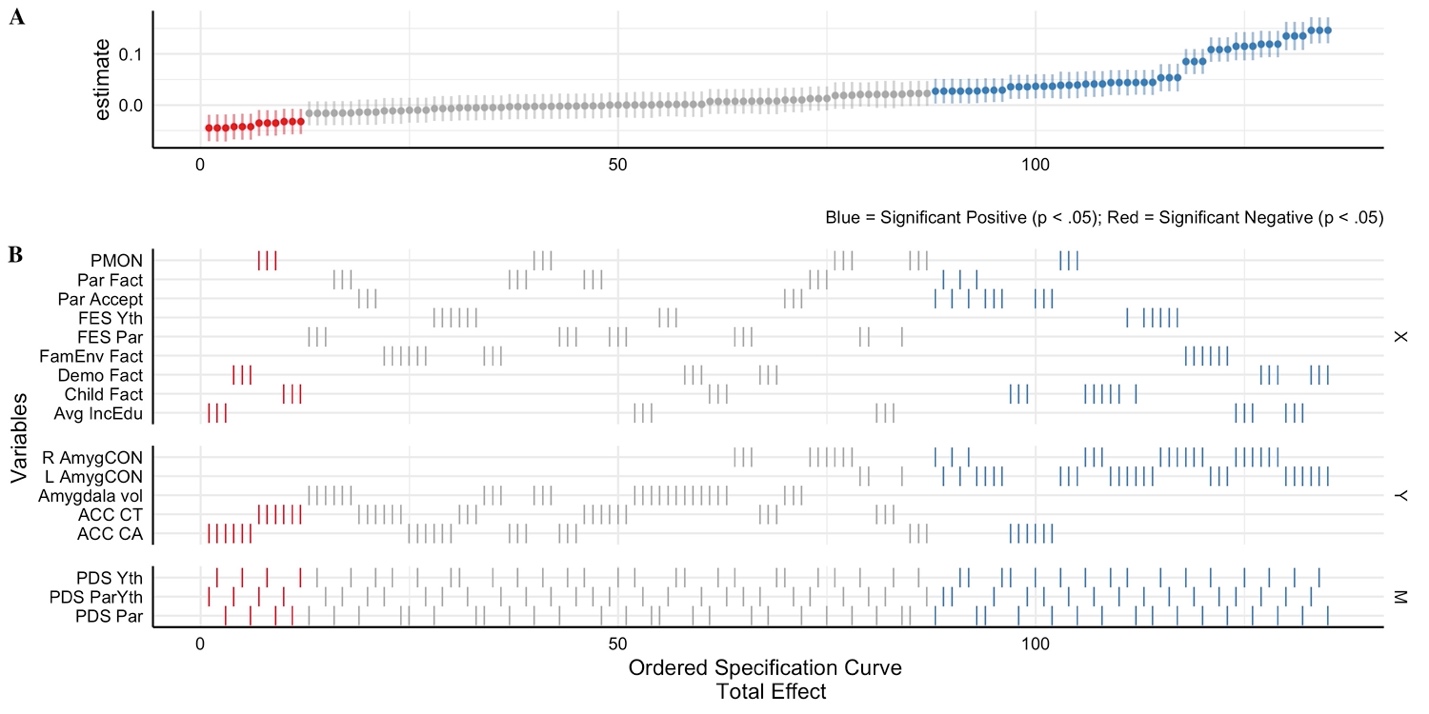


*Figure S6:* Results of the multiverse analysis expressed as specification curves for all of the 135 models. The blue, gray and red colors indicate whether that standardized β estimate was a significant positive (*p* < .05), non-significant (*p* > .05) or a significant negative estimate (*p* < .05), respectively. Age, sex and race were included as covariates in all of the 135 models. **A**. *Total Effect* estimates from mediation models; ordered by size and direction for each estimate for an associated X (predictor), Y (outcome) and M (Mediator). **B**. The associated variables, X, Y and M (Mediator), for each associated effect in the mediation model.

Abbreviations: PMON = Parental Monitoring; Fact = Factor; Par = Parent; Accept = Child Report of Parent Behavior Inventory (i.e., Acceptance); FES = Family Environment Scale (i.e. Conflict; reverse coded); Yth = Youth; FamEnv = Family Environment; Demo = Demographic; Avg IncEdu = Average Parent Reported Income & Education; L/R AmygCON = Left/Right Amygdala Cingulo-Opercular Network connectivity; ACC = Anterior Cingulate Cortex; CT = Cortical Thickness; CA = Cortical Area; PDS = Pubertal Development Scale

Multiverse, **direct effects.**


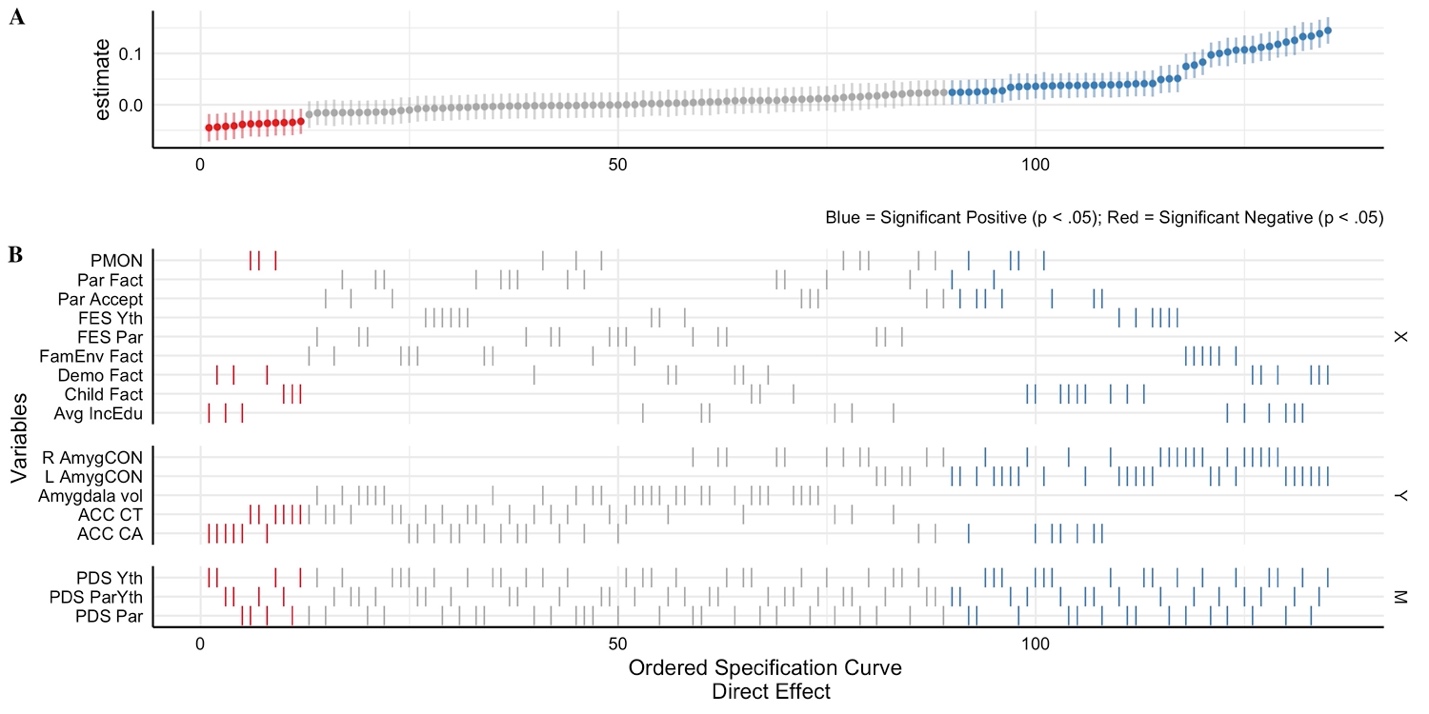


*Figure S7:* Results of the multiverse analysis expressed as specification curves for all of the 135 models. The blue, gray and red colors indicate whether that standardized β estimate was a significant positive (*p* < .05), non-significant (*p* > .05) or a significant negative estimate (*p* < .05), respectively. Age, sex and race were included as covariates in all of the 135 models. **A**. *Direct Effect* estimates from mediation models; ordered by size and direction for each estimate for an associated X (predictor), Y (outcome) and M (Mediator). **B**. The associated variables, X, Y and M (Mediator), for each associated effect in the mediation model.

Abbreviations: PMON = Parental Monitoring; Fact = Factor; Par = Parent; Accept = Child Report of Parent Behavior Inventory (i.e., Acceptance); FES = Family Environment Scale (i.e. conflict); Yth = Youth; FamEnv = Family Environment; Demo = Demographic; Avg IncEdu = Average Parent Reported Income & Education; L/R AmygCON = Left/Right Amygdala Cingulo-Opercular Network connectivity; ACC = Anterior Cingulate Cortex; CT = Cortical Thickness; CA = Cortical Area; PDS = Pubertal Development Scale

Estimates, within factors across a-path, b-path, total effect, direct and indirect effects. **Reference**, Family Environmental Factor (filled squared)


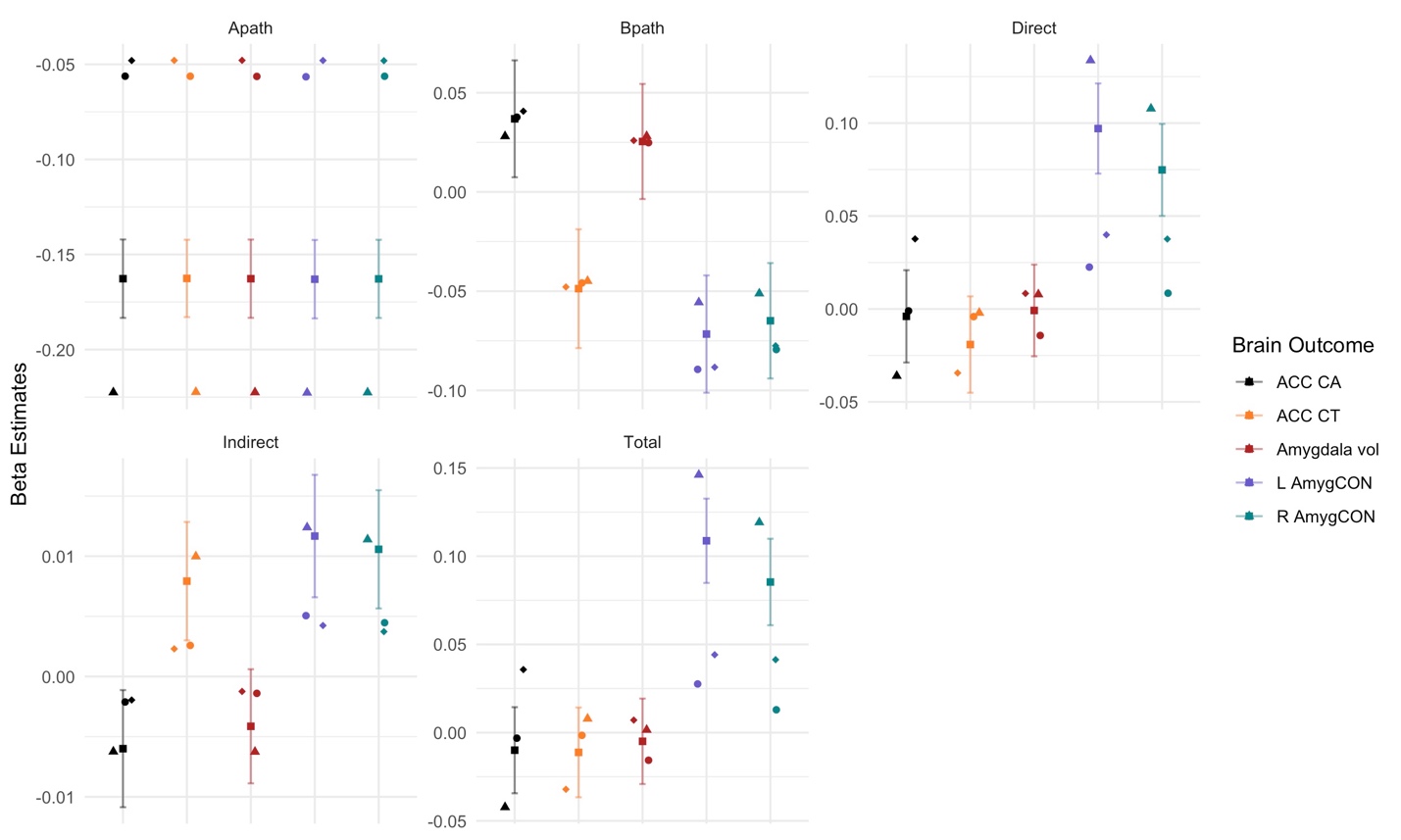


*Figure S8.* Reported standardized β estimates for A-path, B-path, Direct, Indirect and Total effects from the multiverse analyses of *factor derived* scores as IV for Parental Reported Puberty models only. The Family Environment beta and its associated 95% CI is reported**.** ■ = Family Environment; ▲ = Demographic; ● = Parent; ◆ = Child

ACC = Anterior Cingulate Cortex; CA = Cortical Area; CT = Cortical Thickness; Vol = Volume; L/R AmygCON = Left/Right Amygdala Cingulo-Opercular Network Connectivity

Estimates, within individual measures across a-path, b-path, total effect, direct and indirect effects. **Reference**, Family Environmental Factor (filled squared)


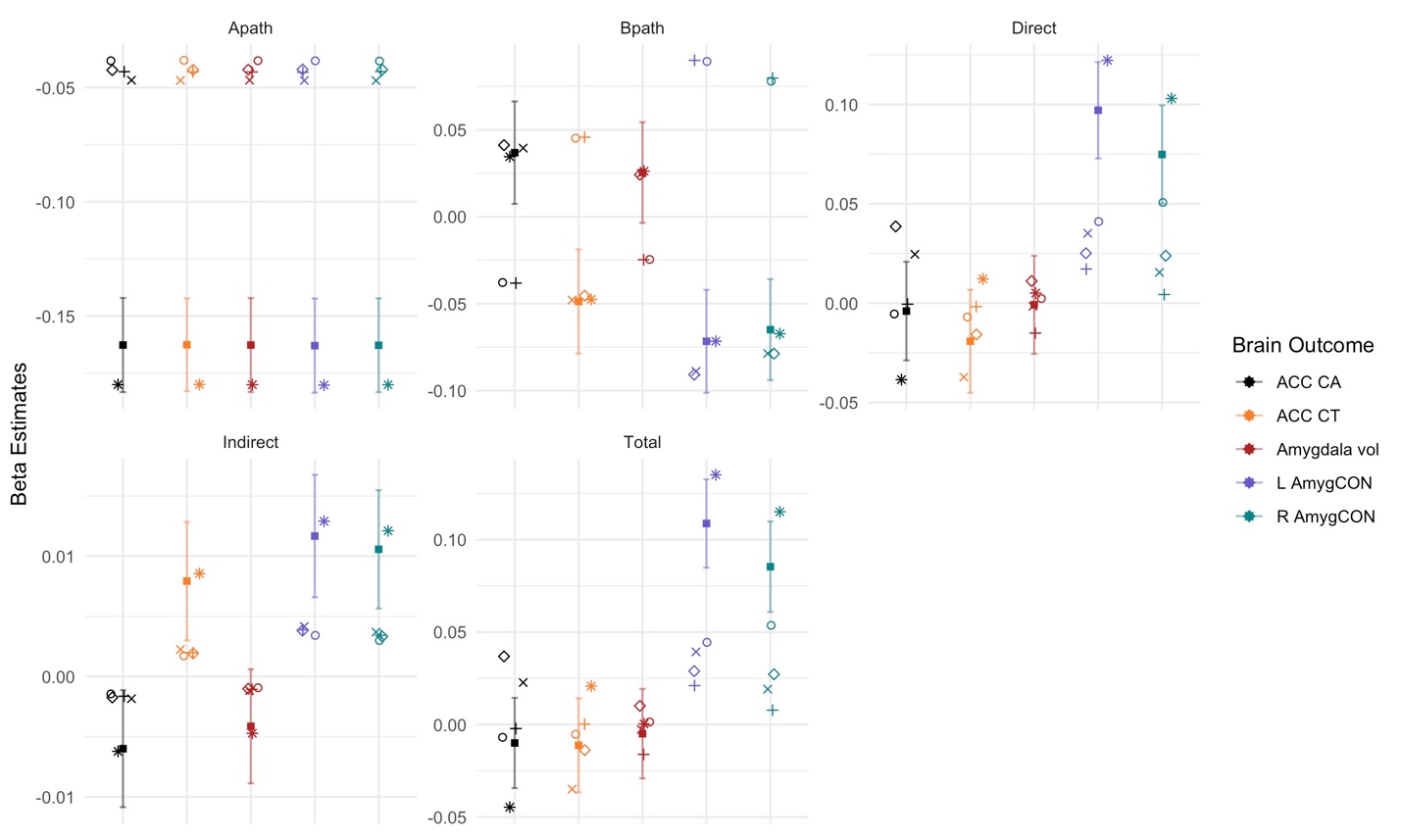


*Figure S9.* Reported standardized β estimates for A-path, B-path, Direct, Indirect and Total effects from the multiverse analyses of *individual measure* scores as IV for Parental Reported Puberty models only. The Family Environment beta and its associated 95% CI is reported. ■ = Family Environment; ○ = FES Youth (i.e., Conflict); + = FES Parent (i.e., Conflict; reverse coded); ✕ = Parental Monitoring; ⬦= Child Report of Parent Behavior Inventory (i.e., Acceptance); * = Avg Income/Education

ACC = Anterior Cingulate Cortex; CA = Cortical Area; CT = Cortical Thickness; Vol = Volume; L/R AmygCON = Left/Right Amygdala Cingulo-Opercular Network Connectivity
